# ARTDeco: automatic readthrough transcription detection

**DOI:** 10.1186/s12859-020-03551-0

**Published:** 2020-05-26

**Authors:** Samuel J. Roth, Sven Heinz, Christopher Benner

**Affiliations:** 1 Bioinformatics and Systems Biology Program, University of California, San Diego, 9500 Gilman Drive, La Jolla, CA 92093-0640 USA; 2 Department of Medicine, University of California, San Diego, 9500 Gilman Drive, La Jolla, CA 92093-0640 USA

**Keywords:** Readthrough transcription, Transcription termination, Transcriptomics, Gene expression, Next-generation sequencing analysis

## Abstract

**Background:**

Mounting evidence suggests several diseases and biological processes target transcription termination to misregulate gene expression. Disruption of transcription termination leads to readthrough transcription past the 3′ end of genes, which can result in novel transcripts, changes in epigenetic states and altered 3D genome structure.

**Results:**

We developed Automatic Readthrough Transcription Detection (ARTDeco), a tool to detect and analyze multiple features of readthrough transcription from RNA-seq and other next-generation sequencing (NGS) assays that profile transcriptional activity. ARTDeco robustly quantifies the global severity of readthrough phenotypes, and reliably identifies individual genes that fail to terminate (readthrough genes), are aberrantly transcribed due to upstream termination failure (read-in genes), and novel transcripts created as a result of readthrough (downstream of gene or DoG transcripts). We used ARTDeco to characterize readthrough transcription observed during influenza A virus (IAV) infection, validating its specificity and sensitivity by comparing its performance in samples infected with a mutant virus that fails to block transcription termination. We verify ARTDeco’s ability to detect readthrough as well as identify read-in genes from different experimental assays across multiple experimental systems with known defects in transcriptional termination, and show how these results can be leveraged to improve the interpretation of gene expression and downstream analysis. Applying ARTDeco to a gene expression data set from IAV-infected monocytes from different donors, we find strong evidence that read-in gene-associated expression quantitative trait loci (eQTLs) likely regulate genes upstream of read-in genes. This indicates that taking readthrough transcription into account is important for the interpretation of eQTLs in systems where transcription termination is blocked.

**Conclusions:**

ARTDeco aids researchers investigating readthrough transcription in a variety of systems and contexts.

## Background

Transcription termination is a fundamental step in gene expression regulation. For most genes, transcription termination is triggered when RNA polymerase II (RNAPII) transcribes a polyadenylation site (PAS) that activates the cleavage and polyadenylation (CPA) complex associated with the C-terminal domain (CTD) of RNAPII [13]. There are two popular models for how CPA recruitment induces transcription termination. In the allosteric model, recruitment of CPA is accompanied by a conformational change in elongating RNAPII, causing dissociation from the DNA and release of the nascent pre-mRNA [36]. In the torpedo model, polyA-dependent cleavage of pre-mRNA by CPA leaves an uncapped nascent RNA emanating from elongating RNAPII. The exonuclease XRN2 degrades the unprotected nascent transcript until it catches up to transcribing RNAPII, causing its release from the DNA [12, 34]. Alternative transcription termination mechanisms have been described for histone genes, snRNAs, and transcripts generated by RNAPI and RNAPIII [11, 21, 25].

**Fig. 1:**
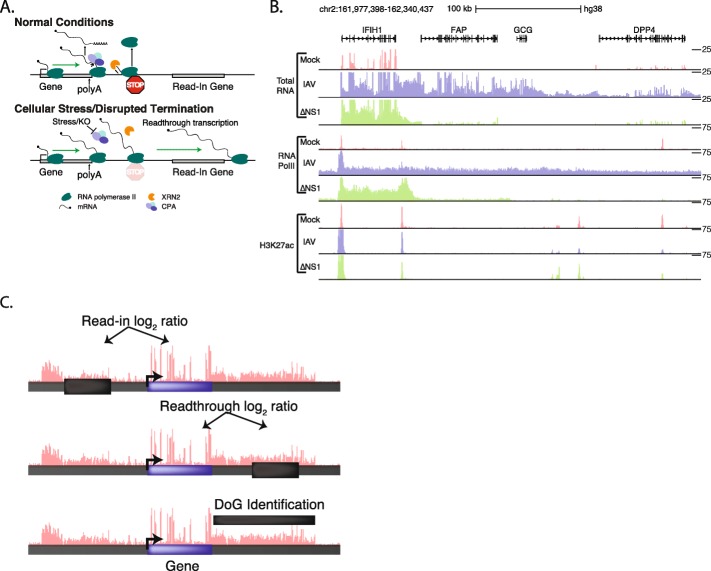
ARTDeco evaluates different aspects of readthrough transcription. **a** Schematic diagram of typical transcription termination (top) and readthrough transcription (bottom). **b** Total RNA-seq, RNA polymerase II ChIP-seq, and H3K27ac ChIP-seq data at *IFIH1* locus. Normalized read coverage ranges are indicated on the right and signals exceeding these levels may be clipped (e.g. RNA-seq coverage on the exons of *IFIH1*). *IFIH1* represents a primary induction gene while *FAP*, *GCG*, and *DPP4* represent read-in genes. **c** Schematic depicting the regions used to quantify read-in levels, readthrough levels, and DoG transcript discovery for each gene

Recent studies have demonstrated that cellular stress can disrupt normal transcription termination, leading to aberrant transcription of intergenic regions downstream of canonical termination sites (termed readthrough transcription or downstream of gene [DoG] transcription) through an unknown mechanism (pictured in Fig. 1a). These stresses include heat shock, osmotic stress, hypoxia, influenza A virus (IAV) infection, herpes simplex virus 1 (HSV-1) infection, senescence, and cancer [3, 4, 6, 9, 10, 18, 26, 31, 32]. In addition to exerting cellular stress, IAV expresses the viral non-structural protein 1 (NS1), which by itself can induce readthrough transcription, presumably by inactivating the poly(A) signal-recognition molecule cleavage and polyadenylation specificity factor (CPSF) 30 [19]. This causes inhibition of CPA activity at poly(A) signal-dependent genes and leads to widespread readthrough transcription [3, 9, 19].

Analyzing gene expression data from samples exhibiting evidence for readthrough transcription poses several challenges: without proper termination, both splicing and polyadenylation of the pre-mRNA may be impaired [37]. Size-selected RNA-sequencing (North-seq) experiments indicate that readthrough/DoG RNAs are long (> 13.5 kb) and not exported from the nucleus [9]. Similarly, HSV-1 infection leads to decreased signal for readthrough transcripts in cytoplasmic RNA relative to both total and nuclear RNA [10]. Ribosome profiling in HSV-1-infected cells indicates that readthrough RNAs are not bound by ribosomes and thus not translated [26]. The observation that readthrough transcription impedes protein expression is important because RNA profiling methods are often used as proxies for gene expression in biomedical research. RNA-seq or microarray profiling in systems with readthrough transcription are therefore likely to provide incorrect estimates of protein levels.

Readthrough transcription can also impact the measurement of gene expression in genes located downstream of sites where transcription termination is inhibited. As aberrant transcription proceeds into downstream genes, RNA templated from these regions may be misinterpreted as evidence for expression of these downstream genes (e.g. *FAP* in Fig. 1b) [9, 26]. Following Rutkowski et al., we will term these loci “read-in” genes. The regulation of read-in genes is easily misinterpreted because the RNAs produced at these loci are unlikely to be exported or translated, and their promoters and other regulatory elements do not regulate their transcript levels. Given that most functional analyses and systems-level studies rely on RNA levels as their primary approach to molecular profiling, this represents a potential source of error when analyzing systems with widespread readthrough transcription. Without correcting for read-in genes, these analyses suffer from the inclusion of aberrantly transcribed read-in genes when studying the molecular pathways and regulatory mechanisms underlying transcriptional responses.

In addition to generating non-canonical and novel transcripts, readthrough transcription can alter the epigenomic state of the genome [4, 9, 10]. In the case of heat shock, osmotic stress, and HSV-1 infection, it has been found that regions exhibiting transcriptional readthrough have increased chromatin accessibility [10, 32]. Strikingly, in IAV infection, transcriptional readthrough causes dynamic changes in 3D genome structure. This phenomenon occurs as elongating RNAPII displaces cohesin, the ring-like complex that spatially constrains the strands of DNA at the base of chromatin loops [9]. In addition, IAV-induced readthrough can result in widespread changes in histone modifications and transcription factor (TF) binding site occupancy [9].

Given the extensive impact that defects in transcription termination and readthrough transcription can have, computational tools are needed to identify and characterize their phenotypes from next-generation sequencing (NGS) profiling data. Although several studies have analyzed readthrough transcription, they have primarily used custom or ad hoc approaches [10, 26, 31, 32, 35]. Presently, there are two published methods designed to analyze readthrough transcription: DoGFinder, a tool that discovers and quantifies intergenic transcripts downstream of genes (DoG transcripts) [10, 26, 31, 32, 35], and DogCatcher, a tool that discovers and quantifies DoGs, Antisense Downstream of Gene (ADoG), Previous of Gene Transcripts (PoGs), and Antisense Previous of Gene (APoG) transcripts in addition to being able to perform differential expression analysis on these transcripts [17]. Both tools provide a useful characterization of readthrough transcription and can aid in the discovery of systems exhibiting transcription termination defects. However, their functionality is limited to searching for aberrant transcripts in intergenic regions.

Here we present Automatic Readthrough Transcription Detection (ARTDeco), a framework for the quantification and characterization of readthrough transcription. ARTDeco expands on the functionality of existing approaches by implementing three separate strategies to quantify readthrough transcription by evaluating (1) the fraction of transcription starting upstream and continuing into a gene (‘read-in level’), (2) the fraction of transcription that continues past the end of genes (‘readthrough level’), and (3) detection of novel DoG transcripts created as a result of readthrough transcription (pictured schematically in Fig. 1c). We assess the performance of ARTDeco on previously generated data for IAV infection and heat shock treatment. We also demonstrate how ARTDeco can be used to quantitatively assess readthrough transcription across large donor datasets and show that eQTLs for read-in genes likely control their upstream gene’s transcription levels. We conclude that our tool is capable of quantifying key features of readthrough transcription to improve the analysis and interpretation of NGS experiments performed on samples with defects in transcription termination.

### Implementation

ARTDeco is written in Python 3.6. It has the following software dependencies: BEDOPS [20], bx-python, DESeq 2[15], HOMER [8], NetworkX [7], NumPy [23], Pandas [16], rpy2, RSeQC [33], and Samtools [14]. Code is available at https://github.com/sjroth/ARTDeco**.**

### ARTDeco analysis framework

ARTDeco requires aligned BAM files, a GTF file of gene annotations, and a chromosome sizes file. Optionally, a metadata file detailing the experimental design and a comparison file detailing the comparisons to be carried out during differential expression analysis can be supplied. The program will quantify expression at genic and intergenic regions (detailed below) and return summary statistics for readthrough transcription and DoG transcripts as well as read-in and readthrough ratios for each gene.

### ARTDeco preprocessing

The input gene annotation (GTF file) is preprocessed into BED files representing the key genomic regions interrogated by ARTDeco. For each gene, all separate isoforms are condensed into a single region starting from the most upstream transcription start site [TSS] to most downstream transcription termination site [TTS]) to avoid misidentifying alternative isoforms as readthrough transcripts. Intergenic regions for detecting read-in and readthrough transcription relative to each gene are then selected as outlined schematically in Fig. 1c and Supplementary Fig. 1b. Genes were excluded from consideration if their annotation fell within another gene. Read-in quantification regions are placed a fixed distance (as defined by the user; 1 kb by default) upstream of the most upstream TSS for each gene to avoid variation in TSS location relative to annotations. Readthrough quantification regions are placed a fixed distance (as defined by the user; 10 kb by default) downstream of each gene to avoid detection of transcription that normally occurs in the region immediately 3′ of the poly(A) signal-dependent cleavage site. The default length of each read-in/readthrough detection region is set to 15 kb (can be user-defined). If another gene is present in the locus, the length of the read-in/readthrough regions are truncated such that they extend a maximum of one-third of the distance to the next gene to avoid detecting signal originating from the other gene. Thus, the length of the read-in and readthrough regions can be expressed as min (maxLength,^1^/_3_*geneDist) where maxLength is the maximum length of a read-in/readthrough region (15 kb by default) and geneDist is the distance to the upstream or downstream gene. The minimum length of both read-in and readthrough regions can be user-defined and is 100 bp by default. If genes are overlapping or too close in proximity, the readthrough/read-in region is removed and not reported for that gene. If one gene falls within the gene body of another gene (as is the case with many small RNAs), that gene is removed from consideration by ARTDeco. Inclusion of these genes leads to issues in interpretation and potential errors due to annotation rather than biological phenomena. Both read-in and readthrough regions are placed into BED files for downstream processing.

### ARTDeco expression quantification

ARTDeco quantifies gene expression (both raw counts and FPKM) using HOMER’s analyzeRepeats.pl and the user-supplied GTF file as well as expression at intergenic regions using HOMER’s annotatePeaks.pl [8]. Expression is quantified across the whole gene body for each transcript in the GTF file and the most highly (maximum) expressed isoform (in FPKM) is stored for downstream processing of read-in and readthrough levels.

### ARTDeco read-in and readthrough level quantification

For each gene, the expression in both raw counts and FPKM for both the maximum isoform of the gene and the intergenic region of interest are grouped together. Then, the log2 ratio of length-normalized counts is computed between the isoform and the read-in/readthrough region (outlined in Fig. 1c and Supplementary Fig. 1b). These ratios define the read-in and readthrough levels for each gene. ARTDeco then infers read-in genes based upon a user-defined threshold for read-in level (0 by default) as well as a user-defined expression threshold level (0.25 FPKM by default) to exclude genes with minimal expression. ARTDeco summarizes the basic statistics of read-in and readthrough levels for the most expressed genes (top 1000 by default).

### ARTDeco gene expression deconvolution

ARTDeco can correct deconvolute the contribution of upstream readthrough transcription to total gene expression by using the upstream read-in expression. In order to do this, it subtracts the length-normalized raw expression in the read-in region from the length-normalized raw gene body expression. If the read-in region has higher expression than the gene body, the gene body expression is set to 0.

### Combining read-in levels with differential expression information

Expression information can be combined with differential expression analysis as performed by DESeq 2[15] to discriminate genes that are directly induced (termed “primary induction”) from those induced as a consequence of read-in transcription from upstream genes (termed “read-in”). This can be useful for enhancing the specificity of the analysis if the experimental condition is expected to impact transcription termination. DESeq2 is carried out on all transcripts in the GTF file as quantified by ARTDeco and this information is combined with read-in ratios for each gene. Genes are thresholded based upon log2 fold change (default is 2), adjusted *p*-value (Benjamini-Hochberg correction as performed by DESeq2; default is 0.05), and expression in FPKM (default is 0.25) and categorized as a primary induction or read-in gene based upon read-in levels (default is 0).

### ARTDeco DoG detection

ARTDeco uses a rolling window approach beginning at the TTS of each gene as defined by our condensed gene annotation. Over each window of the user-specified length (500 bp by default), transcription levels are quantified and the FPKM of the window must meet a user-specified threshold to be considered part of a DoG (0.15 FPKM by default). A DoG can be extended beyond a downstream gene’s TSS if that gene is labeled a read-in gene. After DoGs are discovered for each experiment, their expression is obtained (raw and FPKM). Then, they are combined into a single annotation by taking the union wherein the longest DoG annotation is kept for shared DoGs across experiments. The expression of the unified set of DoGs and their differential expression (if applicable) is also reported (raw and FPKM).

## Results

ARTDeco processes NGS data (e.g., RNA-seq) to characterize the features of readthrough transcription genome-wide. This includes the identification of genes that exhibit transcription downstream of their 3′ ends (readthrough genes), genes that are transcribed as a result of readthrough transcription from upstream genes (read-in genes), as well as detection of novel DoG transcripts created as a result of readthrough transcription. The basic workflow of ARTDeco is detailed in Supplementary Fig. 1a. ARTDeco can work with custom gene annotations and custom genomes. ARTDeco detects read-through events by comparing the levels of transcription in genic and intergenic regions for all genes, evaluating signal both upstream and downstream of genes to distinguish readthrough and read-in events. The intervals used to calculate intergenic transcription levels exclude regions immediately upstream of the transcription start site (TSS, > 1 kb) and downstream of the transcription termination site (TTS, > 10 kb) to avoid detection of RNA signal that arises from incorrect TSS assignment and post-poly(A) site cleavage transcripts that may accumulate during normal termination, respectively. Because closely spaced genes (< 10 kb distance between gene ends) limit the ability to infer intergenic expression levels, these genes are excluded from the analysis. The log_2_ transcript signal ratio of the read-in or readthrough regions versus the gene body expression can be used as a quantification of the degree of readthrough upstream (read-in level) or downstream (readthrough level) of a gene, respectively (Fig. 1c, Supplementary Fig. 1b). In studies where a specific experimental condition is suspected to induce transcription readthrough, ARTDeco can combine its analysis strategy with differential expression analysis to discriminate between genes that are likely regulated by primary induction (i.e. promoter activation) versus read-in genes among all induced genes. ARTDeco also detects unannotated DoG transcripts using a rolling window approach with a minimal FPKM threshold beginning at the TTS of each gene, similar to DoGFinder [35].

### Global quantification of read-through

To evaluate ARTDeco’s ability to quantify transcriptional readthrough across multiple experiments, we analyzed previously generated transcriptomic and epigenetic data from monocyte-derived macrophages infected ex vivo with two strains of IAV as well as a mock infection condition (example of data in Fig. 1b) [9]. The first influenza strain is the highly pathogenic IAV (subtype H5N1) virus (Influenza A/Vietnam/1203/2004 (H5N1) HAlo) used to model severe disease with an intact NS1 protein (called IAV here). The second strain has the same viral genetic background but is mutated to produce a truncated, non-functional NS1 protein (ΔNS1 )[9, 29]. These two strains induce a similar antiviral transcriptional response in the cell, but only IAV infection expresses an intact NS1 protein capable of inhibiting the CPA complex, leading to readthrough transcription. In effect, the ΔNS1 strain allows us to examine antiviral response activation without readthrough while the mock condition has neither antiviral response nor readthrough. This allows us to differentiate antiviral response transcription from readthrough transcription during IAV infection.

**Fig. 2:**
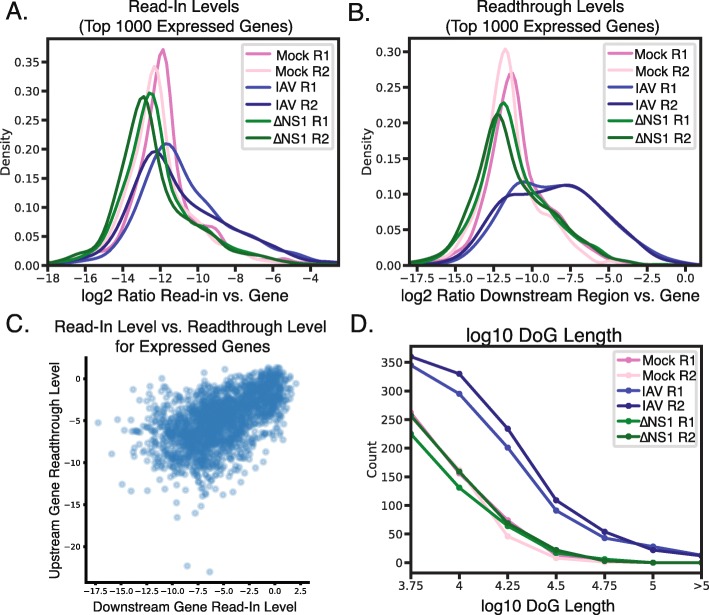
Quantification of readthrough phenotypes in IAV-infected monocyte derived macrophages. **a** Distribution of read-in levels (log2 ratio of reads in read-in region vs. gene body) for top 1000 expressed genes. **b** Distribution of readthrough levels (log2 ratio of reads in downstream region vs. gene body) for top 1000 expressed genes. **c** Downstream gene read-in level vs. upstream gene read-in level in the first replicate of the IAV condition. Both downstream and upstream genes were expressed at a level > 0.25 FPKM (r = 0.55; *p* < 1e-151). **d** Distribution of DoG lengths for DoGs discovered by ARTDeco using default settings (minimum length of 4 kb, window size of 500 bp, and minimum read density of 0.15 FPKM)

First, ARTDeco quantifies the global level of readthrough transcription in each sample, by calculating the genome-wide distributions of read-in and readthrough ratios for the top 1000 expressed genes (Fig. 2a,b). We found that the distributions of both read-in and readthrough ratios were shifted to higher values in the IAV samples relative to both ΔNS1 or mock infection (Fig. 2a,b). Because transcription levels still decay after the cleavage site even when termination is inhibited, readthrough levels, which are measuring the signal produced by readthrough transcription at sites directly downstream of where termination is inhibited, often have a more pronounced signal than read-in levels, which are measured upstream of the next gene, 83,649 bp downstream of the TTS on average. Given that read-in transcription is likely mediated by readthrough transcription from adjacent genes, we quantified this relationship by comparing read-in levels for every expressed gene (> 0.25 FPKM) with the readthrough levels of their upstream gene, finding that these two values were significantly correlated (Fig. 2c, r = 0.55; *p* < 1e-151). This result is quantitatively and qualitatively consistent with the hypothesized relationship between read-in levels and the readthrough levels of the upstream gene. In all, this confirms the ability of ARTDeco to use read-in and readthrough levels to quantify readthrough transcription.

Because read-in levels are defined as the log2 ratio of upstream readthrough transcription to genic transcription, they represent the relative contribution of readthrough to gene expression. Given this observation, we investigated whether read-in levels could potentially aid in deconvoluting the relative contributions of readthrough transcription and canonical gene activation to expression level. We examined all upregulated differentially expressed genes in the IAV condition relative to the mock condition and compared their expression values between IAV and ΔNS1 conditions (Supplementary Fig. 2a). We found that the expression levels between these two datasets was largely correlated (r = 0.72; *p* < 1e-87), however, many genes were expressed more highly in the IAV condition due to read-in transcription (Supplementary Fig. 2a). We then corrected the expression values for both conditions by the estimated fraction of reads due to readthrough and compared their expression. We found that the correlation in gene expression was increased (r = 0.81; *p* < 1e-127) and that this increase was statistically significant (*p* < 0.001; Fisher’s *z* transformation) (Supplementary Fig. 2b). This suggests that the read-in level provides information about the relative contribution of readthrough transcription to gene expression and indicates that ARTDeco can estimate gene expression by removing contributing upstream readthrough.

Another method of quantifying readthrough transcription is the detection of DoG transcripts. Similar to the read-in and readthrough ratios, we performed DoG transcript discovery on mock-, IAV-, and ΔNS1-infected samples (Supplementary Table 2). We found more than twice as many DoGs in IAV-infected samples than the other conditions, consistent with the global increase in readthrough caused by NS1-mediated disruption of transcription termination. Additionally, DoGs found in the IAV condition were much longer than those in the ΔNS1 or mock conditions (almost twice as long on average), which were typically less than 10 kb in length (Fig. 2d).

In order to compare ARTDeco’s ability to detect DoG transcripts to existing methods, we independently used DoGFinder and Dogcatcher to identify DoGs in the IAV condition using default parameters (Supplemental Methods). Despite differences in how transcript detection is performed between the methods, all three methods exhibited comparable sensitivity and detected many of the same DoGs (Supplementary Fig. 3a,b). Notably, Dogcatcher found very few unique DoGs (Supplementary Fig. 3a,b). This is likely because Dogcatcher screens DoGs similarly to DoGFinder (i.e., using a minimum coverage) while maintaining genic reads like ARTDeco. Differentially detected DoGs between the methods are largely explained by technical differences. DoGFinder and Dogcatcher screen DoGs based upon continuous coverage (presence or absence of reads spanning a portion of the screening window). In contrast, ARTDeco extends transcripts based upon a read density threshold measured in FPKM while keeping genic reads. This leads to DoGFinder-specific transcripts in regions with low signal but continuous coverage. Conversely, ARTDeco does not remove genic reads so some DoGs may represent mis-annotation of the TTS or inefficient transcription termination. These methodological differences are reflected by DoGFinder-specific transcripts with lower expression in FPKM (the criteria for ARTDeco) while ARTDeco-specific transcripts have lower per-base coverage (the criteria for DoGFinder) (Supplementary Fig. 3d-e).

In order to validate ARTDeco’s ability to detect DoGs, we looked for independent evidence for transcription of DoGs by examining the levels of H3K36me3 and RNAPII phosphorylated on serine 2 of the CTD (RNAPII S2p) at DoG loci. Both H3K36me3 and RNAPII S2p are associated with transcription elongation, and should be enriched in readthrough regions relative to non-transcribed regions. Because ARTDeco and DoGFinder discovered the most distinct DoGs individually and Dogcatcher discovered very few unique DoGs (only 6; Supplementary Fig. 3b), we chose to compare DoGs from ARTDeco and DoGFinder. We found that DoGs shared between ARTDeco and DoGFinder had comparable occupancy of both signals while DoGs unique to DoGFinder had decreased signal (Supplementary Fig. 3f-g). In summary, we find that ARTDeco has sensitivity comparable to DoGFinder and Dogcatcher and confirmed that the DoGs identified show evidence of transcription elongation.

### Identification of read-in genes

Because pre-mRNAs produced as a result of readthrough transcription are generally not exported from the nucleus and are unlikely to be translated [9, 10, 32], differential RNA levels in samples with readthrough transcription likely misrepresent gene expression levels of newly transcribed genes and may confound functional analyses. Furthermore, readthrough transcription can continue far past the 3′ end of transcribed genes leading to the increase of RNA signal at downstream “read-in” genes. This leads to the illusion that read-in genes are regulated by the biological process being studied. One of the novel functions of ARTDeco is to identify read-in genes to infer whether a given gene is “induced” by readthrough transcription (i.e. read-in) or if it is directly targeted for induction by the cell’s regulatory machinery (referred to here as ‘primary induction’ genes).

**Fig. 3:**
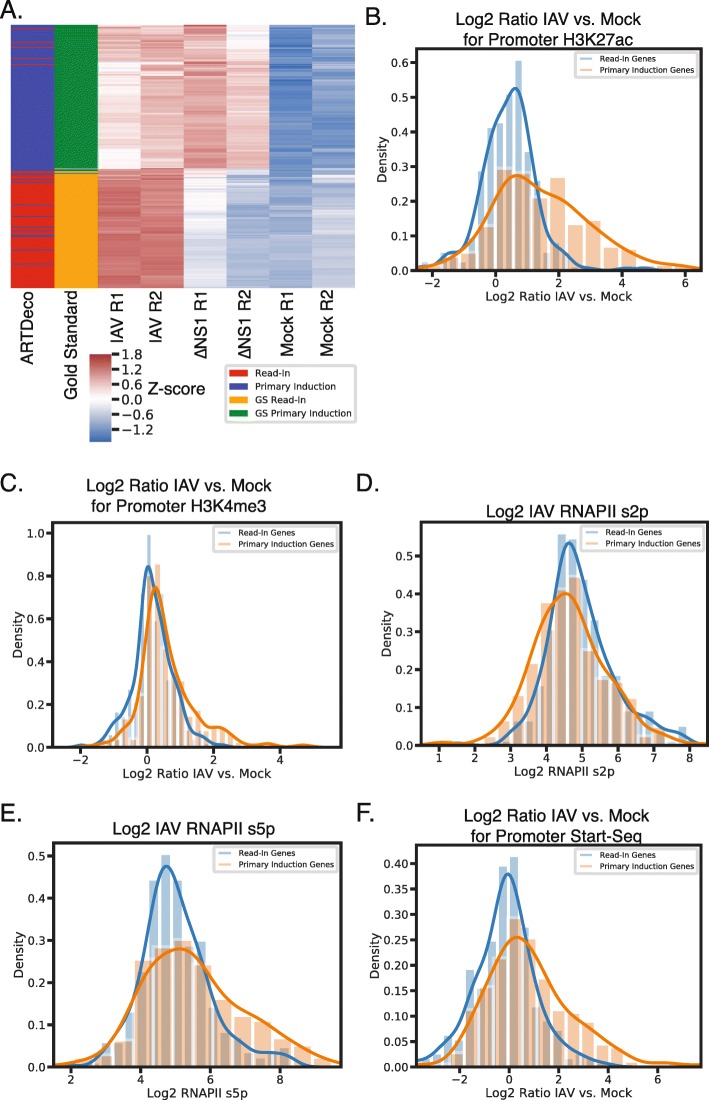
ARTDeco successfully discriminates between genes that are directly induced by IAV infection (primary induction) and genes induced as a results of readthrough transcription (read-in). **a** Heatmap of *z*-normalized expression values and ARTDeco assignments for gold standard primary induction and read-in genes. Thresholds for assigning read-in genes were log2 fold change > 2, adjusted *p*-value < 0.05, and read-in level > − 2. Leftmost column is ARTDeco assignment (blue is primary induction and red is read-in). Next column is gold standard assignment (green is primary induction and gold is read-in). Remaining columns are z-normalized gene expression for IAV replicate 1, IAV replicate 2, ΔNS1 replicate 1, ΔNS1 replicate2, mock replicate 1, and mock replicate 2. **b** Distribution of log2 ratio of H3K27ac for IAV vs. Mock conditions at promoters for primary induction and read-in genes. (*p* < 1e-20; t-test) **c** Distribution of log2 ratio of H3K4me3 for IAV vs. Mock conditions at promoters for primary induction and read-in genes. (*p* < 1e-10; t-test) **d** Distribution of RNA PolII serine-2 phosphorylation (S2p) at promoters in the IAV condition for primary induction and read-in genes. (*p* < 0.001; t-test) **e** Distribution of RNA PolII serine-5 phosphorylation (S5p) in the IAV condition at promoters for primary induction and read-in genes. (*p* < 1e-5; t-test) **f** Distribution of log2 ratio of Start-seq signal for IAV vs. Mock at promoters for primary induction and read-in genes. (*p* < 1e-14; t-test)

We sought to test the ability of ARTDeco to discriminate between primary induction and read-in genes among genes induced by IAV. In order to benchmark our method, we curated a gold standard set of primary induction and read-in genes based on differences in induction in the wild-type IAV and ΔNS1 viruses (Supplemental Methods; Fig. 3b). We considered gold standard primary induction genes to be upregulated in IAV relative to mock infection with clear signs of promoter activation in H3K27ac and RNAPII ChIP-seq data (Supplemental Methods; Supplemental Table 1; example Supplementary Fig. 4a). Similarly, we considered gold standard read-in genes to be upregulated in IAV relative to both mock and ΔNS1 (log2 fold change > 2 and adjusted p-value < 0.05 according to DESeq2) with no signs of promoter activation (Supplemental Methods; Supplemental Table 1; example Supplementary Fig. 4a). In total, there were 163 gold standard primary induction genes and 135 gold standard read-in genes (Supplemental Table 1).

ARTDeco was able to identify IAV primary induction and read-in genes with an F1 score (a measure of the accuracy of classification computed by taking the harmonic mean of the precision and recall; Supplemental Methods) of 0.95 relative to our gold standard. ARTDeco’s performance when inferring read-in genes was robust to different parameters, but optimal when upregulated genes had a log2 fold change > 2, adjusted *p*-value < 0.05 and read-in level > − 2 (for all genes with expression > 0.25 FPKM; number of Gold Standard [GS] Primary Induction Genes = 163, number of GS Read-In Genes = 130, True Positives [TP] = 118, True Negative [TN] = 158, False Positive [FP] = 5, False Negative [FN] = 12) (Supplementary Fig. 4c,d). We also found that ARTDeco was able to infer read-in genes on single experiments without differential expression information and thresholding only on read-in levels (Supplemental Methods; Supplementary Fig. 5a, optimal performance using a read-in level > − 1). Performance was generally poorer when not including differential expression information due to an increase in false positives as reflected in the false discovery rate (FDR) (0.04 with differential expression vs. 0.44 without differential expression) (Supplementary Fig. 5a; F1 = 0.67; GS Primary Induction Genes = 4188, GS Read-In Genes = 128, TP = 105, TN = 4106, FP = 82, FN = 23). One source of false positives were a result of ARTDeco detecting readthrough transcription in the read-in region despite no significant change in genic expression in IAV relative to either mock or ΔNS1 and signs of promoter activation in the downstream gene (ex. *MON2* in Supplementary Fig. 5b). The use of differential expression also helps filter the number of genes considered and, thus, limits potential exposure to errors due to incorrect gene annotations. Based upon this, we conclude that the addition of differential expression allows ARTDeco to improve specificity in experimental designs where readthrough transcription is expected to be regulated in a specific condition.

After using the above parameters (log2 fold change > 2, adjusted p-value < 0.05, and read-in level > − 2) to infer read-in genes with differential expression information, we sought independent validation of our inference. We clustered gene expression profiles for all gold standard genes and found that gene assignments showed expected expression patterns (i.e., true positives [read-in genes] were expressed exclusively in IAV while true negatives [primary induction genes] were expressed in both IAV and ΔNS1 but not in mock) (Fig. 3b). Because read-in genes are transcribed as a result of upstream expression rather than transcription initiation, we hypothesized that promoters of read-in genes would show decreased signs of promoter activation and transcription initiation relative to primary induction genes. As expected, promoters of primary induction genes were enriched for both H3K27ac and H3K4me3 (epigenomic signals associated with promoter activation) in IAV relative to mock while the promoters of read-in genes were not (Fig. 3b,c). Similarly, we examined the phosphorylation state of RNAPII at promoters. Primary induction genes showed higher RNAPII serine-5 phosphorylation (S5p) (a mark of transcription initiation) occupancy at promoters while read-in genes showed higher RNAPII serine-2 phosphorylation (S2p) (a mark of transcription elongation) occupancy (Fig. 3d,e). These data are consistent with the hypothesis that the promoters of primary induction genes are activated by IAV while the promoters of read-in genes are not.

In order to assess whether the promoters of primary induction genes showed more evidence of transcription initiation than those of read-in genes, we also examined Start-seq data at promoters in both IAV- and mock-infected THP-1 cells (a human monocytic cell line) [9]. Start-seq captures newly initiating short RNAs that approximate rates of transcription initiation at TSSs [27]. We observed increased signals of transcription initiation at promoters of primary induction genes as compared to read-in genes despite differences in cell type (Fig. 3f). This further strengthens the conclusion that primary induction genes represent a stimulus-specific response while read-in genes are expressed due to upstream readthrough transcription rather than promoter activation. In all, these data show that ARTDeco is able to discriminate between primary induction and read-in genes in a set of differentially expressed genes.

### Functional analysis of primary induction and read-in genes

**Fig. 4:**
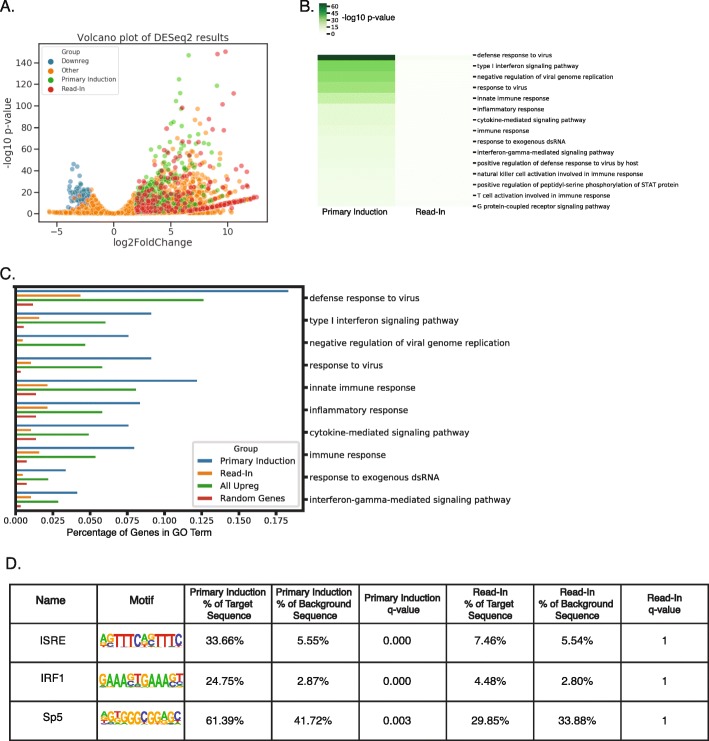
Read-in genes mainly contribute noise to downstream functional analysis of differentially regulated genes. **a** Volcano plot of DESeq2 results for the maximum expressed isoform for each gene from IAV-infected macrophages vs. Mock-infected controls. Genes were considered up- or down-regulated if they had |log2 fold change| > 2 and adjusted p-value < 0.05 as well as FPKM > 0.25. **b** Heat map of GO enrichment (−log10 p-value) for top 15 GO terms in primary induction and read-in genes. **c** Bar chart of proportion of genes from each gene list in a given GO term for top 10 GO terms for primary induction genes, read-in genes, all upregulated genes, and random 500 genes with expression > 0.25 FPKM. **d** HOMER motif enrichments (q-value) for top 3 known motifs in primary induction genes

Read-in genes represent over half (301/545) of all upregulated genes despite not being directly activated by IAV infection (Fig. 4a). Given these read-in genes are not directly targeted for activation by the host transcriptional machinery and likely not expressed as proteins, it is possible that these genes represent biological noise and could dilute the results of functional analyses. With this in mind, we assessed the impact of read-in genes on common functional analyses such as gene ontology (GO) enrichment [2]. Assessing GO enrichment separately on primary induction and read-in genes, we found that primary induction genes were strongly enriched for GO terms consistent with viral defense and immune response. In contrast, read-in genes showed minimal evidence for GO term enrichment, consistent with the hypothesis that read-in genes represent transcriptional noise. (Fig. 4c). We also compared these enrichments with the GO enrichment for all upregulated genes, finding that inclusion of read-in genes did not identify additional enriched GO terms and diluted the fraction of regulated genes in each of the enriched terms relative to just analyzing the primary induction genes (Fig. 4c). Given that GO is incomplete and has known biases such as method of investigation, curation practices, and authorship, it is possible that read-in genes are not properly functionally annotated [1, 30]. With this in mind, we analyzed the TF binding motifs in the promoters of primary induction and read-in genes, reasoning that promoter sequences directly activated by the infection should be enriched for binding motifs for TFs activated during viral infection. We performed motif-finding using HOMER and found that promoters of primary induction genes were enriched for interferon-stimulated response elements (ISRE) while promoters of read-in genes lacked significant enrichment for TF binding motifs (Fig. 4d). Together, our findings suggest that read-in genes are not directly activated as part of the immune response to infection and therefore should be excluded from functional or regulatory element analysis when attempting to infer regulatory mechanisms or functional responses in systems with readthrough transcription.

### Extension of ARTDeco to other experimental systems and NGS data types

**Fig. 5:**
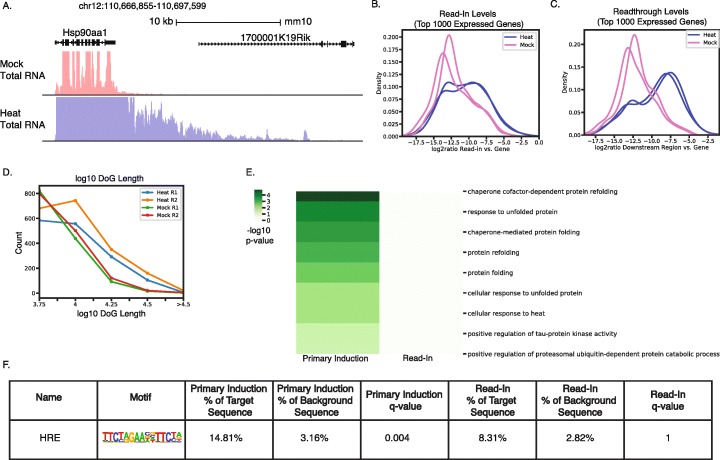
ARTDeco analysis of readthrough transcription induced by heat shock in NIH 3T3 cells. **a** Total RNA-seq levels at the *Hsp90aa1* locus in mouse fibroblasts for heat shock and mock conditions from Vilborg et al. [32]. *Hsp90aa1* represents the primary induction genes while *1700001K19Rik* is a read-in gene defined by ARTDeco. **b** Distribution of read-in levels for top 1000 expressed genes following heat shock. **c** Distribution of readthrough levels for top 1000 expressed genes following heat shock. **d** Distribution of DoG lengths in both mock and heat shock conditions. **e** Heat map of GO term enrichment (−log10 p-value) for top 15 enriched GO terms for primary induction and read-in genes. **f** HOMER motif enrichment for primary induction and read-in genes

In order to validate ARTDeco on non-IAV datasets, we reanalyzed data from heat shock-treatment of NIH 3T3 cells [32], another stimulus known to induce transcriptional readthrough (Fig. 5a). Similar to IAV data, we observed that all global signals of readthrough were elevated (i.e., distribution of read-in/readthrough level, DoG length, and DoG expression) (Fig. 5b-d). Next, we assigned primary induction and read-in genes for the heat shock data. Similar to IAV, for primary induction genes we found significant GO term and TF motif enrichment that was consistent with a heat shock response while no significant enrichment was found for read-in genes (Fig. 5e-f). These results demonstrate that ARTDeco can successfully identify transcriptional readthrough and define primary and read-in gene sets in additional datasets, using the optimized default parameters determined in IAV infection.

In order to demonstrate the flexibility and general applicability of ARTDeco to different experimental data types, we applied it to two methods that assess transcription by measuring RNAPII engagement: RNAPII ChIP-seq and mNET-seq. RNAPII ChIP-seq directly measures DNA binding of the RNAPII complex, while mNET-seq measures nascent transcripts that are associated with the RNAPII complex [22]. First, we applied ARTDeco to RNAPII ChIP-seq data from IAV, ΔNS1-, and mock-infected cells (Supplementary Fig. 6a). Consistent with previous analyses, the distribution of readthrough levels reflects a defect in termination present in IAV infected samples but not the other two conditions, similar to the results generated using total RNA-seq, despite the different data type (Fig. 2b, Supplementary Fig. 6a). Additionally, we found that total RNA-seq data was robust to different downstream readthrough distances while RNAPII ChIP-seq was not (Fig. 2a, Supplementary Fig. 6a-c). Distributions of readthrough levels with a 5 kb distance were more similar between conditions and readthrough was therefore harder to detect on a global level compared to analysis using a 10 kb distance (Supplementary Fig. 6a-b). Thus, ARTDeco’s default parameter of a 10 kb downstream readthrough distance is flexible with respect to data type.

Next, we applied ARTDeco to a published data set that used mNET-seq to profile transcription in response to influenza infection (IAV H1N1 WSN/33, IAV H1N1 Puerto Rico/8/34, IAV H3N2 Udorn/72, IAV H3N2 Udorn/72: NS1Δ99, and Influenza B virus [IBV] Florida/04/2006) as well as an siRNA construct for the CPSF complex, salt shock treatment using KCl, and inducible expression of wild-type and mutant NS1 proteins [3]. Consistent with their reported results, we found that cells infected with influenza virus, subjected to KCl treatment, or deficient in the CPSF complex had higher readthrough levels relative to cells in the mock condition, reflecting decreased transcription termination efficiency. Interestingly, we confirmed the presence of readthrough transcription in IAV H3N2, which contains a deletion in the NS1 protein (Supplementary Fig. 6d). This is consistent with the hypothesis of Bauer et al. [3] that cellular stress may drive part of the readthrough phenotype in A549 and HEK293 cells. In summary, we show that ARTDeco is compatible with multiple NGS data types with different characteristics.

### Reinterpretation of eQTLs identified in data with readthrough transcription

**Fig. 6:**
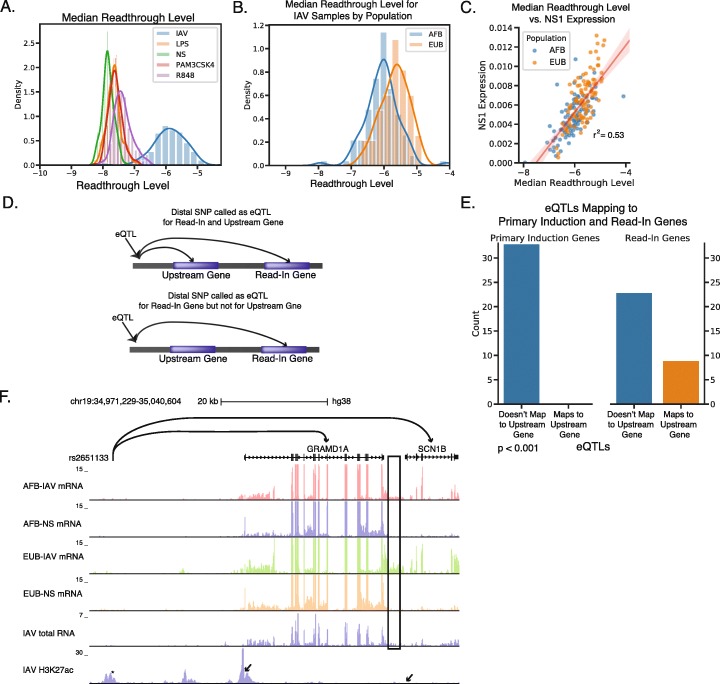
ARTDeco analysis of donor monocytes infected with IAV reveals that eQTLs mapping to read-in genes also frequently map to upstream genes. **a** Distribution of median readthrough levels for top 1000 expressed genes for all samples from Quach et al. [24]. Grouped by treatment condition. **b** Distribution of median readthrough levels for top 1000 expressed genes for IAV samples from Quach et al. [24]. Grouped by population of origin. **c** Scatter plot comparing median readthrough level of top 1000 expressed genes with proportion of reads mapping to IAV NS1 gene (r^2^ = 0.53, *p* < 1e-33). **d** Schematic of two eQTL assignments that are difficult to interpret when readthrough transcription is present. On the top, a SNP is assigned as an eQTL for both the upstream gene and the read-in gene. On the bottom, a SNP located in the upstream gene is assigned as an eQTL for the read-in gene only. The first case represents eQTLs that may modulate the expression of the read-in gene by changing the expression of the upstream gene. **e** Bar chart showing the number of eQTLs mapped by Quach et al. [24] to genes assigned as read-in and primary induction genes. eQTLs are classified as either mapping to the upstream gene as outlined in 6B or not mapping to the upstream gene. Enrichment was computed using Fisher exact test (*p* < 0.001). **f** Example of an eQTL (rs2661133) mapped by Quach et al. [24] that maps to both a read-in gene (*SCN1B*) and the upstream gene (*GRAMD1A*) in IAV-infected samples. Genome browser tracks corresponding to mRNA from an African Belgian (AFB) and a European Belgian (EUB) from IAV-infected and non-stimulated (NS) conditions as well as total RNA and H3K27ac for IAV infection from Heinz et al. [9]. The readthrough region is outlined in the black box

To demonstrate how ARTDeco can improve the analysis of large-scale datasets that exhibit signs of readthrough transcription, we used ARTDeco to reanalyze RNA-seq profiles from primary human monocytes derived from 200 individual donors. Within the original study, monocytes from each donor were genotyped and infected with IAV (H1N1 strain A/USSR/90/1977) or stimulated with lipopolysaccharide (LPS), Pam3CSK4, or R848 in vitro to elicit innate immune responses with the goal of mapping expression quantitative trait loci (eQTLs) [24]. We assessed the presence of readthrough transcription in these datasets by quantifying the median readthrough level of the top 1000 expressed genes as a summary statistic for samples from each donor in each condition. This analysis revealed that IAV-infected samples showed significantly greater median readthrough ratios relative to the other stimuli profiled, consistent with the expected inhibition of transcription termination in samples infected with IAV (Fig. 6a).

While analyzing IAV samples, we observed that some samples generally had higher levels of readthrough transcription than others, prompting us to consider whether ARTDeco could be used to quantitatively assess differences in global readthrough across samples. For example, samples from donors of European origin (EUB) had significantly higher median readthrough ratios than samples from donors of African ancestry (AFB) (Fig. 6b, *p* < 1e-8, t-test), suggesting readthrough ratios may offer a quantitative estimate of the degree to which transcription termination is impacted by infection. In order to corroborate these observations, we compared the median readthrough level from each sample to the expression of viral NS1 RNA in each sample, finding the values to be highly correlated (Fig. 6c, r^2^ = 0.53, *p* < 1e-33). NS1 mRNA levels are likely correlated with other aspects of infection, including the efficiency of viral entry, viral replication rates, and antiviral host responses, and it was noted in the original study that AFB samples showed higher expression of immune response genes such as chemokines and cytokines and thus were likely more resistant to infection [24]. However, given the fact that NS1 is both necessary and sufficient to inhibit transcription termination [3, 9], the correlation between readthrough transcription levels and NS1 expression is consistent with the molecular functions of the viral protein.

In view of the widespread evidence for readthrough transcription in the IAV-infected samples, we hypothesized that eQTLs that map to genes aberrantly transcribed by readthrough transcription (i.e. read-in genes) may be regulating transcription in upstream regions rather than directly controlling transcription activation of the eQTL-associated read-in gene (Fig. 6d). Using our list of inferred primary induction and read-in genes, we reexamined eQTLs (as inferred in the original analysis) defined in IAV-infected conditions. We hypothesized that eQTLs mapping to read-in genes would also map to upstream genes that serve as the source of readthrough transcription, while eQTLs mapping to primary induction genes would be more likely to map near or within the gene itself. We found that 9/32 (28%) of eQTLs mapping to ARTDeco-defined read-in genes also mapped to their upstream genes, while none of the eQTLs mapping to primary induction genes also mapped to their upstream genes (Fig. 6e, *p* < 1e-3, Fisher’s Exact Test, Supplementary Table 3). For example, in the case of the read-in gene *SCN1B*, the SNP rs2651133 was also assigned as eQTL to its upstream gene, *GRAMD1A*, in the IAV condition (Fig. 6f). This SNP falls near a promoter-distal enhancer upstream of *GRAMD1A*, where it likely influences regulatory mechanisms such as TF binding or promoter-enhancer interactions to modulate the activity of *GRAMD1A*. Since the promoter of *SCN1B* lacks epigenetic evidence for activation after IAV infection (Fig. 6f, bottom), it is likely that the same eQTL affects the expression of *SCN1B* by directly modulating the expression of *GRAMD1A*, which then leads to readthrough transcription into the *SCN1B* locus. These findings underscore the need to be careful when interpreting the functions of eQTLs in the presence of readthrough transcription.

## Discussion

Here we present ARTDeco, a framework for comprehensively characterizing and quantifying readthrough transcription from NGS data. ARTDeco globally quantifies the degree of readthrough transcription using read-in levels, readthrough levels, and detection of DoG transcripts. We demonstrate that the medians of the read-in and readthrough level distributions for the top-expressed genes represent useful summary statistics for characterizing the degree of readthrough in a given sample. These measures represent a novel advance in the detection of readthrough transcription. ARTDeco expands upon existing methods for DoG transcript discovery by allowing the discovered transcripts to extend into annotated gene bodies to avoid arbitrary truncation [17, 35]. This allows for a more precise quantification of readthrough as well as more representative transcripts from large regions of transcriptional readthrough that extend through multiple genes (Fig. 1b). ARTDeco’s approach is robust to multiple data types including RNA-seq, mNET-seq, and RNAPII ChIPseq (Figs. 2, 6, Supplementary Fig. 6) making it a versatile tool for the characterization and detection of transcriptional readthrough. Additionally, it requires less preprocessing and has a nearly 2-fold faster runtime than DoGFinder and a nearly 5-fold faster runtime than Dogcatcher (Supplemental Methods; Table 1). ARTDeco’s flexibility and performance in addition to its novel measures of readthrough transcription represent a significant advance in analytical tools for studying defects in transcription termination.

**Table 1:** Run time comparison for DoGFinder and ARTDeco

Task	Number of Runs	Average Run Time (s)
ARTDeco Full	10	1095.76
ARTDeco DoG Mode	10	982.83
Dogcatcher Preprocessing	10	1307.25
Dogcatcher (no differential expression)	10	4085.60
Dogcatcher (with differential expression)	10	4593.81
DoGFinder Preprocessing	10	982.71
DoGFinder	10	1065.85

In addition to global quantification of readthrough transcription, ARTDeco provides per-gene quantification. This provides an opportunity to study readthrough at the level of single genes in the context of both downstream readthrough and upstream read-in. The quantification of read-in levels can also enable the deconvolution of gene expression in systems with transcriptional readthrough. Additionally, each method of readthrough quantification enables us to pinpoint loci of interest in order to study the effects of readthrough on the epigenome and genome structure. Many of the mechanisms of how these changes occur are still unclear. For example, change in genome 3D structure due to transcriptional readthrough has been noted in both IAV infection and heat shock [4, 9]. Using readthrough levels and DoG transcripts, we may be able to better characterize the specific loci that are affected. This would lend great insight into how the mechanism of transcription induces these changes in genome 3D structure and epigenetic regulation.

An open question is what determines the level of readthrough. Work in HSV-1 infection suggests that sequence context at the TTS is a more important determinant of readthrough than expression level [10]. ARTDeco’s quantification of readthrough levels could potentially lend insight to this and hint at potential mechanisms. Additionally, it has been posited that readthrough has an effect on the expression of downstream genes via mechanisms such as transcriptional interference [5, 28]. It remains unclear to what degree this impacts transcriptional regulation and gene expression writ large. Quantification of read-in level allows us to more directly measure these effects by elucidating the relationship between upstream readthrough transcription and gene expression.

A novel function of ARTDeco is the identification of read-in genes. To our knowledge, it is the first software tool that is designed to characterize this phenomenon. This is important as many functional analyses rely on gene expression levels to make inferences (e.g., differential expression, co-expression, etc.) and read-in genes represent a potential source of noise when employing these techniques. We demonstrated the ability to confidently identify read-in genes from NGS profiling data, and showed that these genes likely represent noise in functional analysis when analyzing differentially regulated genes in two different conditions (IAV and heat shock). Our analyses underscore the advantage of treating these genes as noise rather than a potential false signal in the data.

We showed that in a population study of transcriptional responses to IAV infection that a significant proportion of eQTLs mapping to read-in genes also mapped to genes upstream (Fig. 6c,d). In these cases, readthrough transcription is the probable mechanism by which the eQTL influences expression for variants mapped to read-in genes. Given the known difficulty of both mapping and interpreting the functional impact of these SNPs, it is important to correct for transcriptional readthrough when studying gene expression variation in populations in the context of systems with disrupted transcription termination. Our findings suggest that readthrough transcription analysis should be routinely incorporated into population-scale analyses of systems that may contain readthrough in order to better interpret eQTLs.

## Conclusions

Readthrough transcription is an emergent phenotype that has been characterized in several systems including IAV infection, HSV-1 infection, heat shock, salt stress, senescence and renal carcinoma [3, 4, 6, 9, 10, 18, 26, 31, 32]. Given its relative novelty, it is likely that more stresses cause defects in transcription termination, and this phenotype may be more common than previously thought. The use of median readthrough level for top expressed genes as a summary statistic greatly aids discovery of these stresses. Further, ARTDeco can be used to analyze systems where components of the transcription termination machinery are knocked out in order to further analyze mechanisms of termination. In all, ARTDeco will aid future researchers by providing a systematic characterization of readthrough transcription.

## Availability and requirements

**Project name:** ARTDeco.

**Project home page:** https://github.com/sjroth/ARTDeco

**Operating system(s):** Platform independent.

**Programming language:** Python.

**Other requirements:** Python 3.6, BEDOPS 2.4 or higher, bx-python 0.8 or higher, DESeq2 1.2 or higher, HOMER 4.9 or higher, NetworkX 2.2 or higher, NumPy 1.16 or higher, Pandas 0.24 or higher, rpy2 2.9, RSeQC 3.0 or higher, and Samtools 1.9 or higher.

**License:** MIT License.

**Any restrictions to use by non-academics:** No restrictions.

## Supplementary information

**Additional file 1 Supplementary Figure 1**: (a) Basic flowchart of ARTDeco functions. Program inputs are BAM files, a GTF file, and a chromosome sizes file as well as optional inputs for differential expression modes comprised of a meta file and a comparisons file. Data files are preprocessed into HOMER tag directories, a condensed gene annotation BED, and intergenic (read-in and downstream) BED files. From here, ARTDeco can compute read-in and readthrough statistics (left branch) or detect DoGs. Read-in levels for genes are used for DoG transcript discovery (details in Methods). (b) Schematic depicting the regions used to quantify read-in levels, readthrough levels, and DoG transcript discovery for each gene (maxlen is 15 kb by default). Examples of each region and total RNA-seq levels during IAV infection are depicted for the *IFIH1* locus. **Supplementary Figure 2**: Deconvolution of gene expression for upregulated genes in IAV relative to mock. (a) Uncorrected expression for IAV replicate 1 and ΔNS1 replicate 1. (r = 0.72; *p* < 1e-77) (b) Corrected expression for IAV replicate 1 and ΔNS1 replicate 1. (r = 0.81; *p* < 1e-127). **Supplementary Figure 3**: Assessment of Downstream of Gene (DoG) transcripts. (a) Total RNAseq and H3K27ac ChIPseq at the *IFIH1* locus and DoGs identified by ARTDeco and DoGFinder. (b) Venn diagram of all DoGs called by ARTDeco and DoGFinder using both IAV replicates using default coverage parameters and a sliding window of 500 bp. (c) Distribution of DoG lengths for DoGs called by ARTDeco and DoGFinder. (d) Distribution of RNA-seq FPKM values for DoGs identified by ARTDeco and DoGFinder. (e) Distribution of RNA-seq read coverage for DoGs identified by ARTDeco and DoGFinder. (f) Log2 FPKM H3K36me3 occupancy for DoGs assigned by ARTDeco and DoGFinder as well as random regions. (g) Log2 FPKM RNAPII s2p occupancy for DoGs assigned by ARTDeco and DoGFinder as well as random regions. **Supplementary Figure 4**: Examples of primary induction and read-in genes from IAV-infected macrophages. (a) Example of a gold standard true positive (read-in) gene (*RNF144A*). Gene expression is upregulated in IAV relative to ΔNS1 and mock with low (> 0.5 FPKM) expression in ΔNS1. Additionally, there are no RNA PolII and H3K27ac ChIP-seq peaks (as called by HOMER) at the promoter regions. (b) Example of gold standard true negative (primary induction) gene (*TNFSF13B*). Gene expression is upregulated in IAV and ΔNS1 relative to mock. Additionally, there are both RNA PolII and H3K27ac peaks (as called by HOMER) at the promoter region indicating transcription initiation. (c) Benchmarking of ARTDeco performance for inference of read-in genes using false positive rate (FPR), false negative rate (FNR), false discovery rate (FDR), and F1 score while varying DESeq2 log2 fold change. Values for adjusted p-value, FPKM, and read-in level are 0.05, 0.25 and 0, respectively. (d) Benchmarking for ARTDeco performance for inference of read-in genes using FPR, FNR, FDR, and F1 score while varying read-in level. Values for log2 fold change, adjusted p-value, and FPKM are 2, 0.05, and 0.25, respectively. **Supplementary Figure 5**: Evaluation of read-in gene identification without using a control condition. (a) Benchmarking for ARTDeco performance for inference of read-in genes without differential expression while varying read-in level. Gene expression is > 0.25 FPKM. (b) Example of a gene (*MON2*) that was marked as a read-in gene despite being initiated. There is substantial readthrough originating from the upstream gene *USP15*. **Supplementary Figure 6**: Analysis of RNAPII ChIP-seq and mNet-seq data using ARTDeco. (a) Distribution of readthrough levels for IAV, ΔNS1, and mock for top 1000 expressed genes based on ARTDeco’s analysis of RNAPII ChIP-seq data (instead of RNA-seq data) using the default 10 kb downstream readthrough distance. (b) Distribution of readthrough levels for IAV, ΔNS1, and mock for top 1000 expressed genes based on ARTDeco’s analysis of RNAPII ChIP-seq data using a 5 kb downstream readthrough distance. (c) Distribution of readthrough levels for IAV, ΔNS1, and mock for top 1000 expressed genes based on ARTDeco’s analysis of total RNA-seq data using a 5 kb downstream readthrough distance. (d) Distribution of readthrough levels for mNET-seq data from Bauer et al. [3] for top 1000 expressed genes. Cell types are denoted in legend as A549 and HEK293. Treatment conditions are as follows: IAV H1N1 WSN/33, IAV H1N1 Puerto Rico/8/34, IAV H3N2 Udorn/72, IAV H3N2 Udorn/72: NS1Δ99, Influenza B virus [IBV] Florida/04/2006, KCl, wildtype and mutant NS1 proteins, siLUC, and siCPSF. Conditions where readthrough was observed in the original analysis conducted by Bauer et al. [3] have distribution curves with higher opacity.

**Additional file 2.** Supplementary Table 1: List of primary induction and read-in genes identified in IAV-infected macrophages.

**Additional file 3.** Supplementary Table 2: DoGs discovered by ARTDeco for IAV-, ΔNS1-, and mock- infected macrophages. Genomic coordinates are relative to the hg38 version of the human genome.

**Additional file 4.** Supplementary Table 3: List of IAV-associated eQTLs mapped to primary induction and read-in genes.

**Additional file 5 Supplementary Methods.**

## Data Availability

Data from Heinz et al. [9] was obtained from GEO accession GSE103477 (available at https://www.ncbi.nlm.nih.gov/geo/query/acc.cgi?acc=GSE103477). Data from Vilborg et al. [32] was obtained from GEO accession GSE98906 (available at https://www.ncbi.nlm.nih.gov/geo/query/acc.cgi?acc=GSE98906). Data from Bauer et al. [3] was obtained from NCBI SRA SRP132032 (available at https://trace.ncbi.nlm.nih.gov/Traces/sra/?study=SRP132032). Data from Quach et al. [24] was obtained from the EGA accession EGAS00001001895 (available at https://www.ebi.ac.uk/ega/studies/EGAS00001001895).

## Abbreviations

ARTDeco: Automatic Readthrough Transcription Detection
IAV: Influenza A virus
RNAPII: RNA polymerase II
PAS: Polyadenylation site
CPA: Cleavage and polyadenylation complex
CPSF: Cleavage and polyadenylation specificity factor
CTD: C-terminal domain of RNAPII
eQTL: Expression quantitative trait locus
NS1: Non-structural protein 1
HSV: Herpes simplex virus
DoG: Downstream of Gene
TSS: Transcription start site
TTS: Transcription termination site
FPKM: Fragments Per Kilobase of transcript per Million mapped reads
GO: Gene ontology
