# Supplementary material for: ARTDeco: automatic readthrough transcription detection
Source: BMC Bioinformatics. 2020 May 26;21:214. doi: 10.1186/s12859-020-03551-0 (PMC7249449; doi:10.1186/s12859-020-03551-0)
Supplement: Supplementary file 1 — Additional file 1 Supplementary Figure 1: (a) Basic flowchart of ARTDeco functions. Program inputs are BAM files, a GTF file, and a chromosome sizes file as well as optional inputs for differential expression modes comprised of a meta file and a comparisons file. Data files are preprocessed into HOMER tag directories, a condensed gene annotation BED, and intergenic (read-in and downstream) BED files. From here, ARTDeco can compute read-in and readthrough statistics (left branch) or detect DoGs. Read-in levels for genes are used for DoG transcript discovery (details in Methods). (b) Schematic depicting the regions used to quantify read-in levels, readthrough levels, and DoG transcript discovery for each gene (maxlen is 15 kb by default). Examples of each region and total RNA-seq levels during IAV infection are depicted for the IFIH1 locus. Supplementary Figure 2: Deconvolution of gene expression for upregulated genes in IAV relative to mock. (a) Uncorrected expression for IAV replicate 1 and ΔNS1 replicate 1. (r = 0.72; p < 1e-77) (b) Corrected expression for IAV replicate 1 and ΔNS1 replicate 1. (r = 0.81; p < 1e-127). Supplementary Figure 3: Assessment of Downstream of Gene (DoG) transcripts. (a) Total RNAseq and H3K27ac ChIPseq at the IFIH1 locus and DoGs identified by ARTDeco and DoGFinder. (b) Venn diagram of all DoGs called by ARTDeco and DoGFinder using both IAV replicates using default coverage parameters and a sliding window of 500 bp. (c) Distribution of DoG lengths for DoGs called by ARTDeco and DoGFinder. (d) Distribution of RNA-seq FPKM values for DoGs identified by ARTDeco and DoGFinder. (e) Distribution of RNA-seq read coverage for DoGs identified by ARTDeco and DoGFinder. (f) Log2 FPKM H3K36me3 occupancy for DoGs assigned by ARTDeco and DoGFinder as well as random regions. (g) Log2 FPKM RNAPII s2p occupancy for DoGs assigned by ARTDeco and DoGFinder as well as random regions. Supplementary Figure 4: Examples of primary induction and read-in genes from [file 12859_2020_3551_MOESM1_ESM.pdf]

# Figure 1

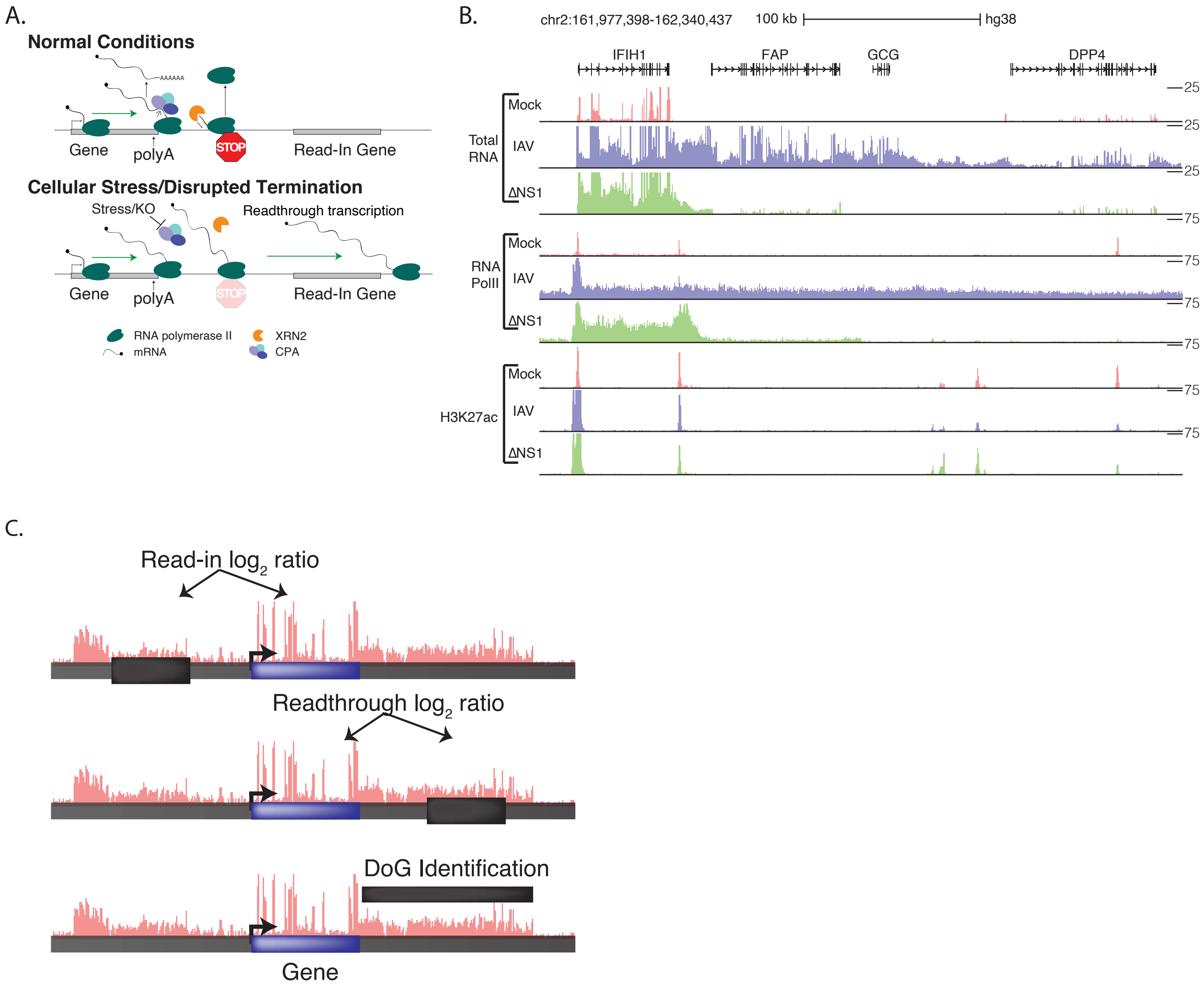

A.
Normal Conditions
AAAAAA
STOP
Gene
polyA
Read-In Gene
Cellular Stress/Disrupted Termination
Stress/KO
Readthrough transcription
STOP
Gene
polyA
Read-In Gene
RNA polymerase II
mRNA
XRN2
CPA
B.
chr2:161,977,398-162,340,437
100 kb
hg38
IFIH1
FAP
GCG
DPP4
Total RNA
Mock
IAV
ΔNS1
25
25
25
RNA PolII
Mock
IAV
ΔNS1
75
75
75
H3K27ac
Mock
IAV
ΔNS1
75
75
75
C.
Read-in log2 ratio
Readthrough log2 ratio
DoG Identification
Gene

Figure 2

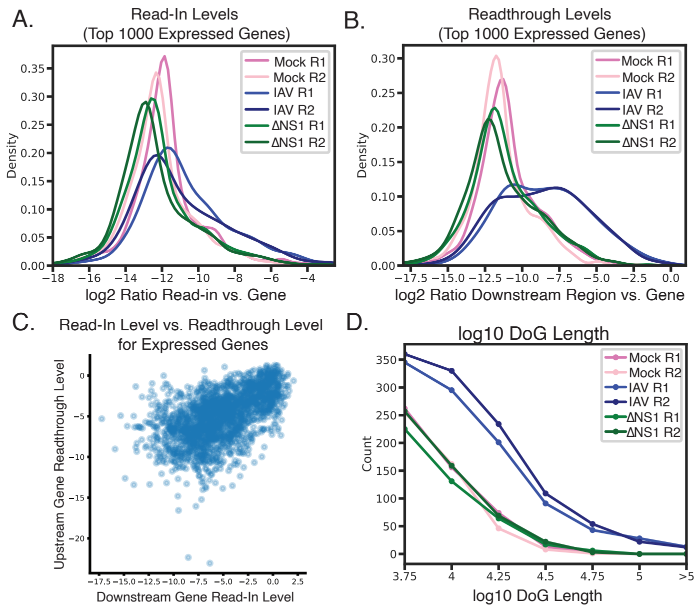
A.
Read-In Levels
(Top 1000 Expressed Genes)
Mock R1
Mock R2
IAV R1
IAV R2
ΔNS1 R1
ΔNS1 R2
Density
log2 Ratio Read-in vs. Gene
B.
Readthrough Levels
(Top 1000 Expressed Genes)
Density
log2 Ratio Downstream Region vs. Gene
C.
Read-In Level vs. Readthrough Level
for Expressed Genes
Upstream Gene Readthrough Level
Downstream Gene Read-In Level
D.
log10 DoG Length
Count
log10 DoG Length

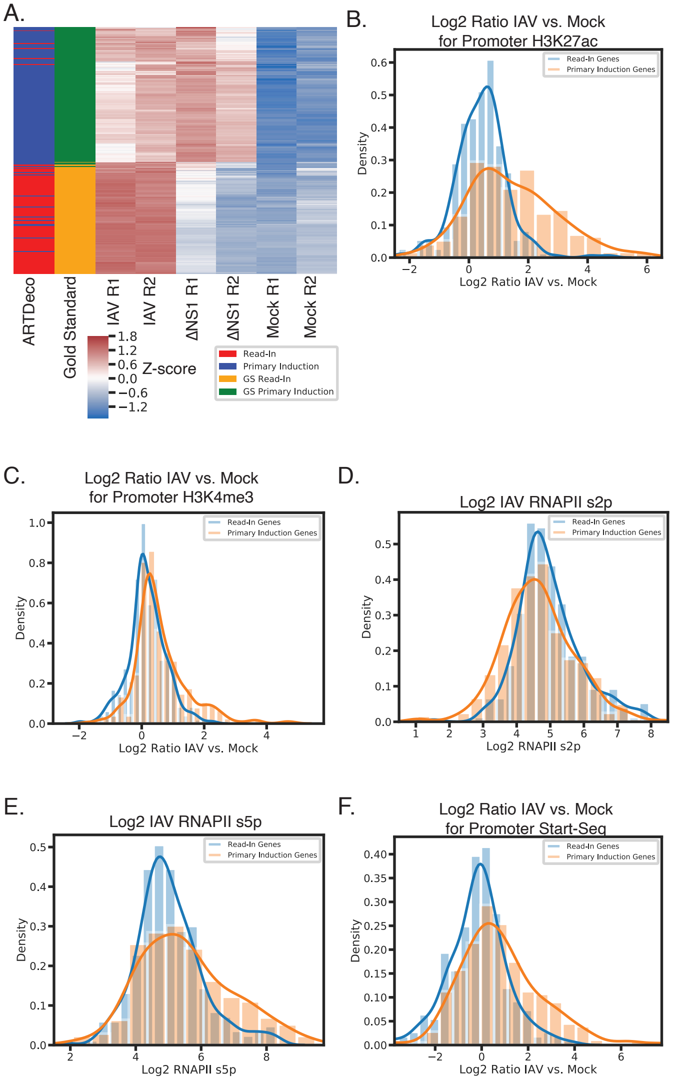

A.
ARTDeco
Gold Standard
IAV R1
IAV R2
ΔNS1 R1
ΔNS1 R2
Mock R1
Mock R2
Z-score
Read-In
Primary Induction
GS Read-In
GS Primary Induction
B.
Log2 Ratio IAV vs. Mock for Promoter H3K27ac
Read-In Genes
Primary Induction Genes
Density
Log2 Ratio IAV vs. Mock
C.
Log2 Ratio IAV vs. Mock for Promoter H3K4me3
Read-In Genes
Primary Induction Genes
Density
Log2 Ratio IAV vs. Mock
D.
Log2 IAV RNAPII s2p
Read-In Genes
Primary Induction Genes
Density
Log2 RNAPII s2p
E.
Log2 IAV RNAPII s5p
Read-In Genes
Primary Induction Genes
Density
Log2 RNAPII s5p
F.
Log2 Ratio IAV vs. Mock for Promoter Start-Seq
Read-In Genes
Primary Induction Genes
Density
Log2 Ratio IAV vs. Mock

Figure 4

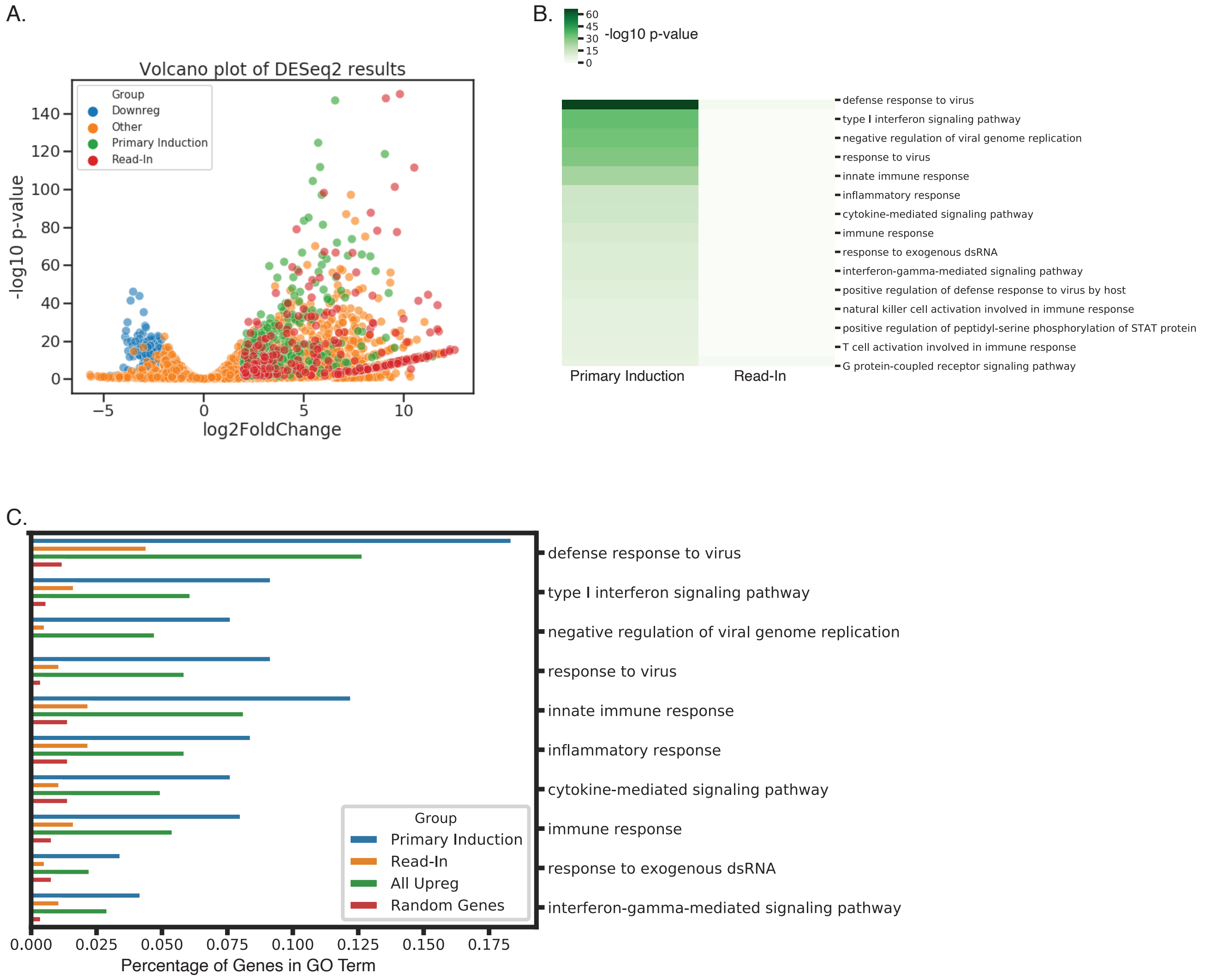


D.

| Name | Motif | Primary Induction % of Target Sequence | Primary Induction % of Background Sequence | Primary Induction q-value | Read-In % of Target Sequence | Read-In % of Background Sequence | Read-In q-value |
|---|---|---|---|---|---|---|---|
| ISRE | 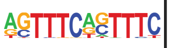 | 33.66% | 5.55% | 0.000 | 7.46% | 5.54% | 1 |
| IRF1 | 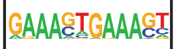 | 24.75% | 2.87% | 0.000 | 4.48% | 2.80% | 1 |
| Sp5 | 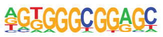 | 61.39% | 41.72% | 0.003 | 29.85% | 33.88% | 1 |

Figure 5

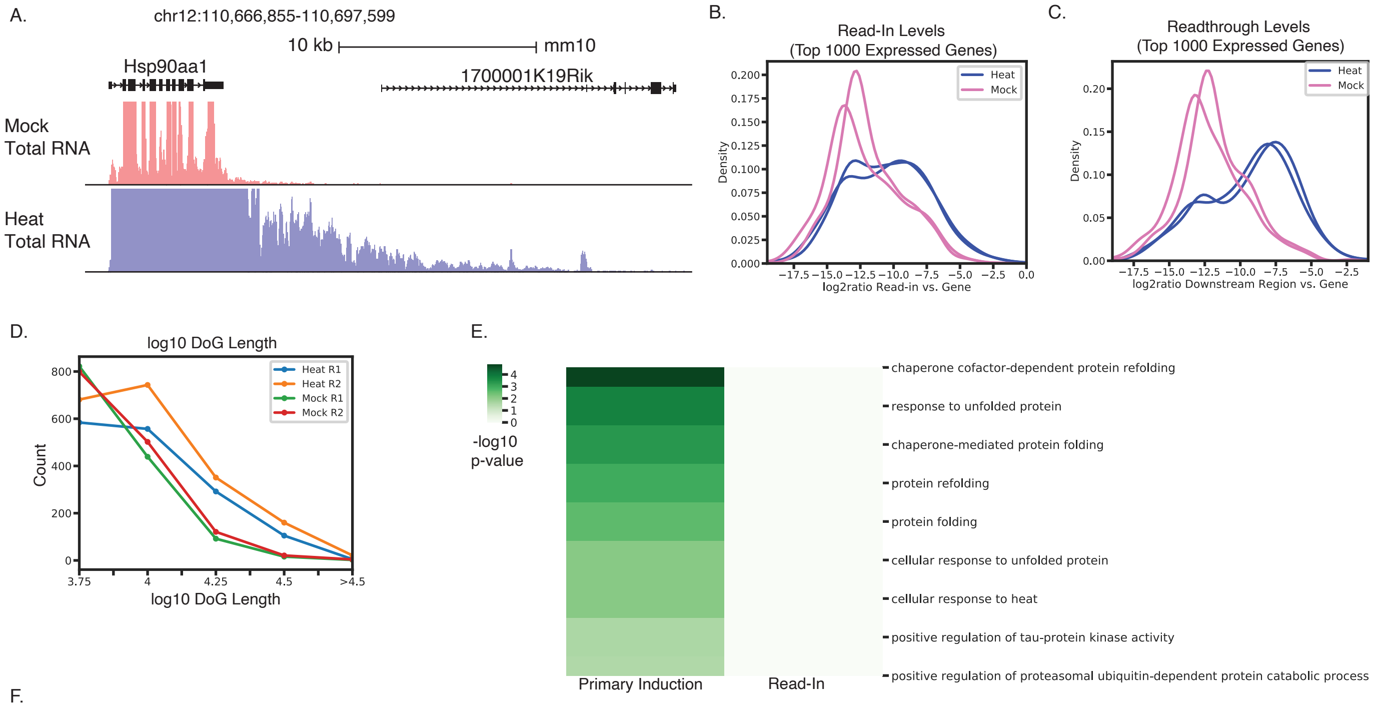


F.

| Name | Motif | Primary Induction % of Target Sequence | Primary Induction % of Background Sequence | Primary Induction q-value | Read-In % of Target Sequence | Read-In % of Background Sequence | Read-In q-value |
|---|---|---|---|---|---|---|---|
| HRE | TTCTAGAA[illegible]TTCTA | 14.81% | 3.16% | 0.004 | 8.31% | 2.82% | 1 |

Figure 6

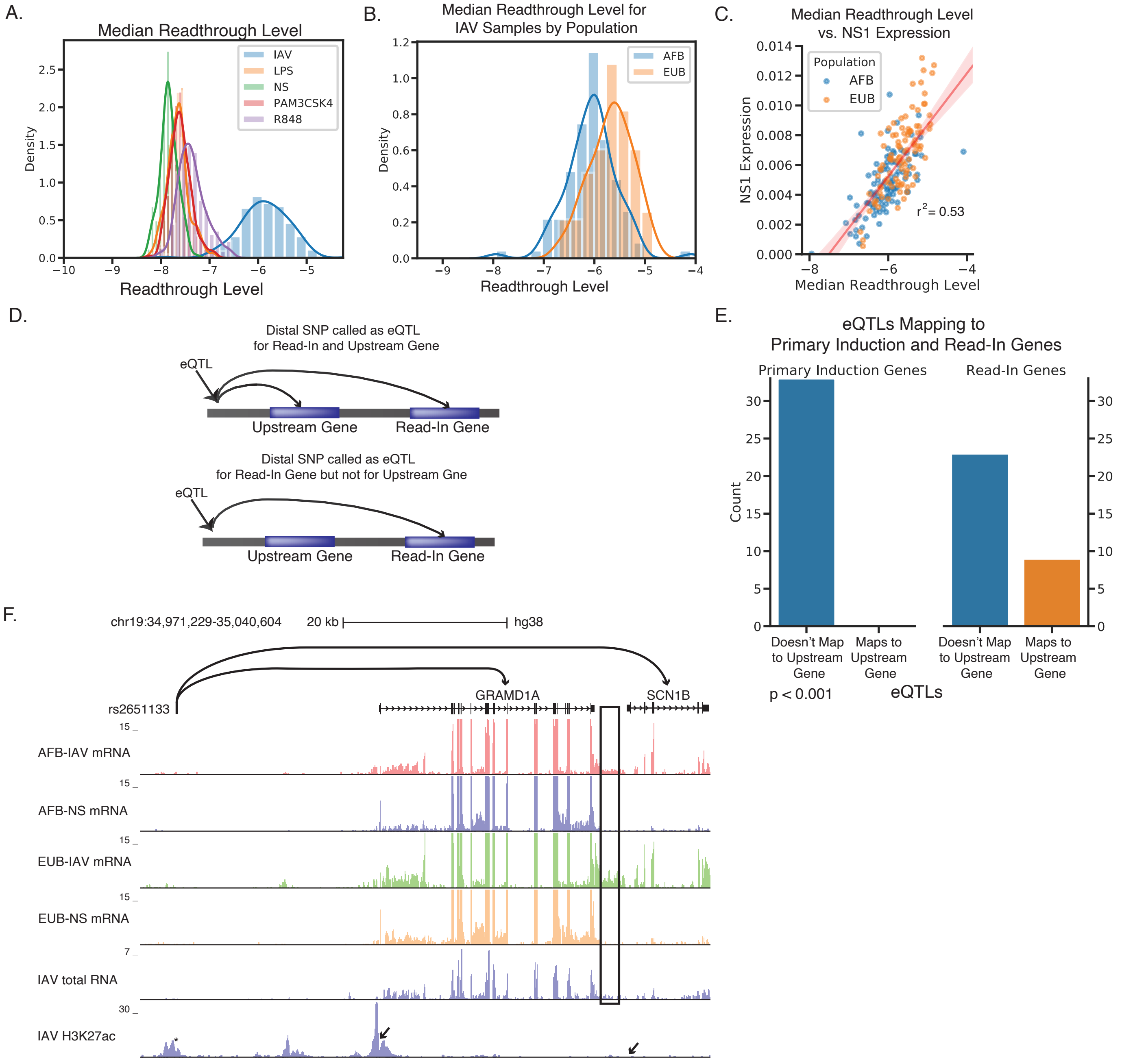

A.
Median Readthrough Level
IAV
LPS
NS
PAM3CSK4
R848
Density
Readthrough Level
B.
Median Readthrough Level for
IAV Samples by Population
AFB
EUB
Density
Readthrough Level
C.
Median Readthrough Level
vs. NS1 Expression
Population
AFB
EUB
NS1 Expression
r2= 0.53
Median Readthrough Level
D.
Distal SNP called as eQTL
for Read-In and Upstream Gene
eQTL
Upstream Gene
Read-In Gene
Distal SNP called as eQTL
for Read-In Gene but not for Upstream Gne
eQTL
Upstream Gene
Read-In Gene
E.
eQTLs Mapping to
Primary Induction and Read-In Genes
Primary Induction Genes
Read-In Genes
Count
Doesn't Map to Upstream Gene
Maps to Upstream Gene
Doesn't Map to Upstream Gene
Maps to Upstream Gene
p < 0.001
eQTLs
F.
chr19:34,971,229-35,040,604
20 kb
hg38
rs2651133
GRAMD1A
SCN1B
AFB-IAV mRNA
AFB-NS mRNA
EUB-IAV mRNA
EUB-NS mRNA
IAV total RNA
IAV H3K27ac

Figure S1

A.

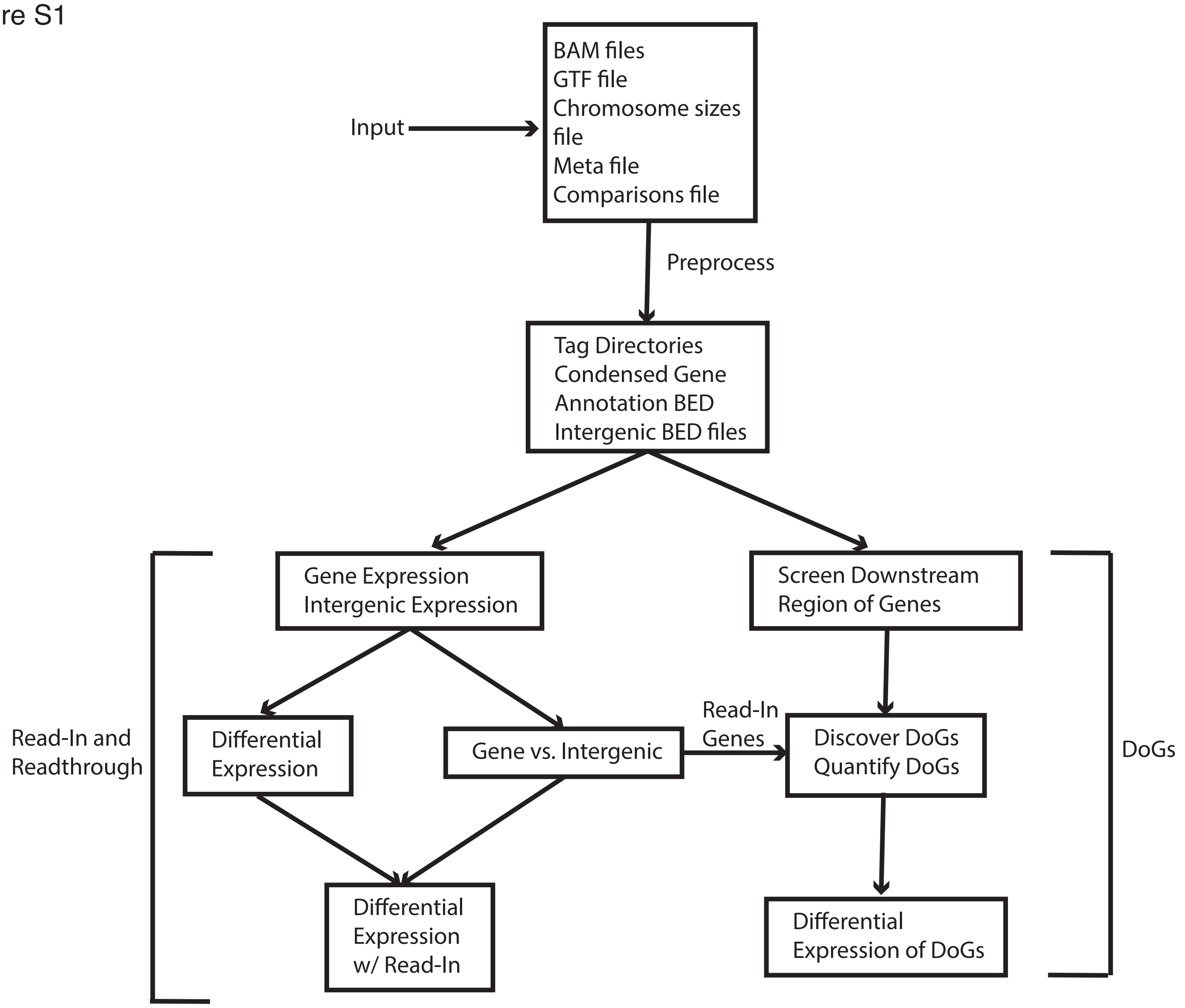
Input
BAM files
GTF file
Chromosome sizes file
Meta file
Comparisons file
Preprocess
Tag Directories
Condensed Gene Annotation BED
Intergenic BED files
Gene Expression
Intergenic Expression
Screen Downstream Region of Genes
Read-In and Readthrough
Differential Expression
Gene vs. Intergenic
Read-In Genes
Discover DoGs
Quantify DoGs
DoGs
Differential Expression w/ Read-In
Differential Expression of DoGs

B.

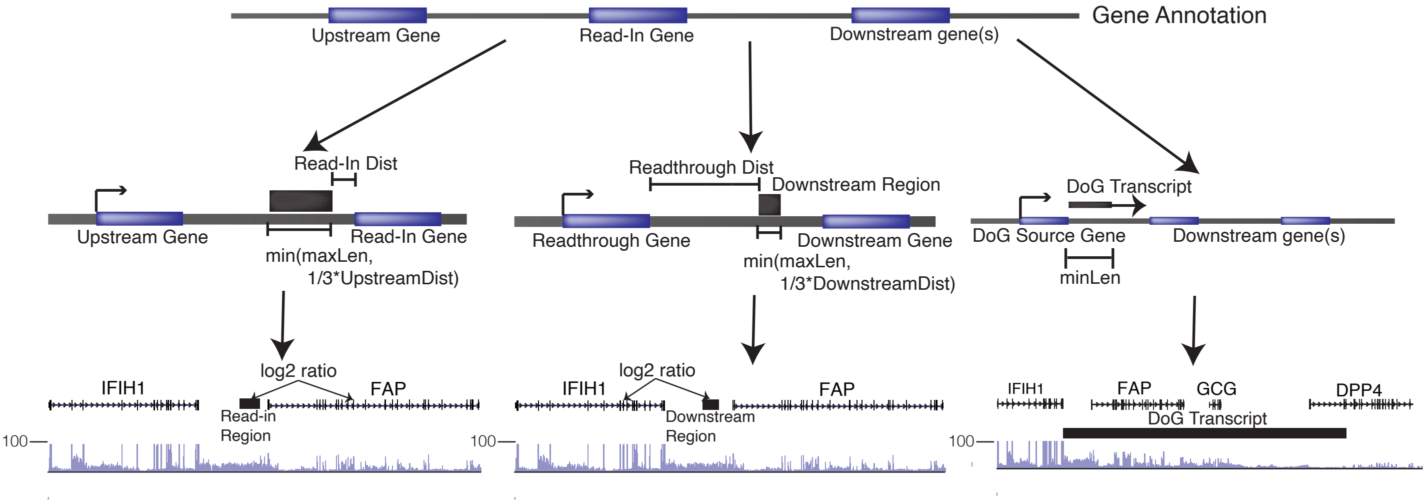
Gene Annotation
Upstream Gene
Read-In Gene
Downstream gene(s)
Read-In Dist
Upstream Gene
Read-In Gene
min(maxLen, 1/3*UpstreamDist)
Readthrough Dist
Downstream Region
Readthrough Gene
Downstream Gene
min(maxLen, 1/3*DownstreamDist)
DoG Transcript
DoG Source Gene
Downstream gene(s)
minLen
IFIH1
log2 ratio
FAP
Read-in Region
100
IFIH1
log2 ratio
FAP
Downstream Region
100
IFIH1
FAP
GCG
DPP4
DoG Transcript
100

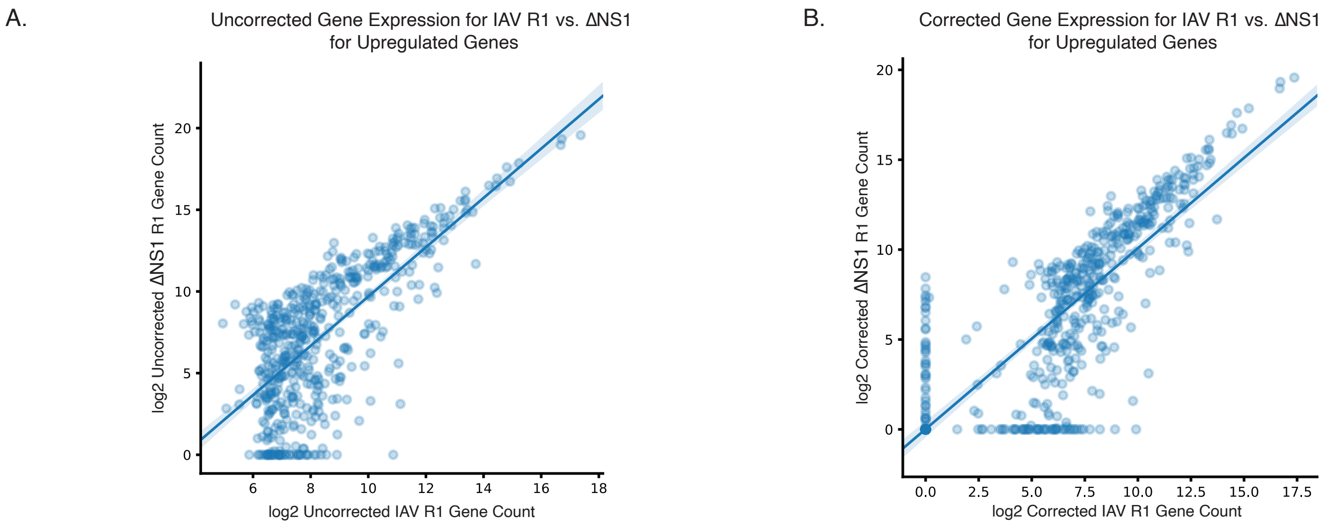

A.
Uncorrected Gene Expression for IAV R1 vs. ΔNS1 for Upregulated Genes
log2 Uncorrected ΔNS1 R1 Gene Count
log2 Uncorrected IAV R1 Gene Count
0
5
10
15
20
6
8
10
12
14
16
18
B.
Corrected Gene Expression for IAV R1 vs. ΔNS1 for Upregulated Genes
log2 Corrected ΔNS1 R1 Gene Count
log2 Corrected IAV R1 Gene Count
0
5
10
15
20
0.0
2.5
5.0
7.5
10.0
12.5
15.0
17.5

# Figure S3

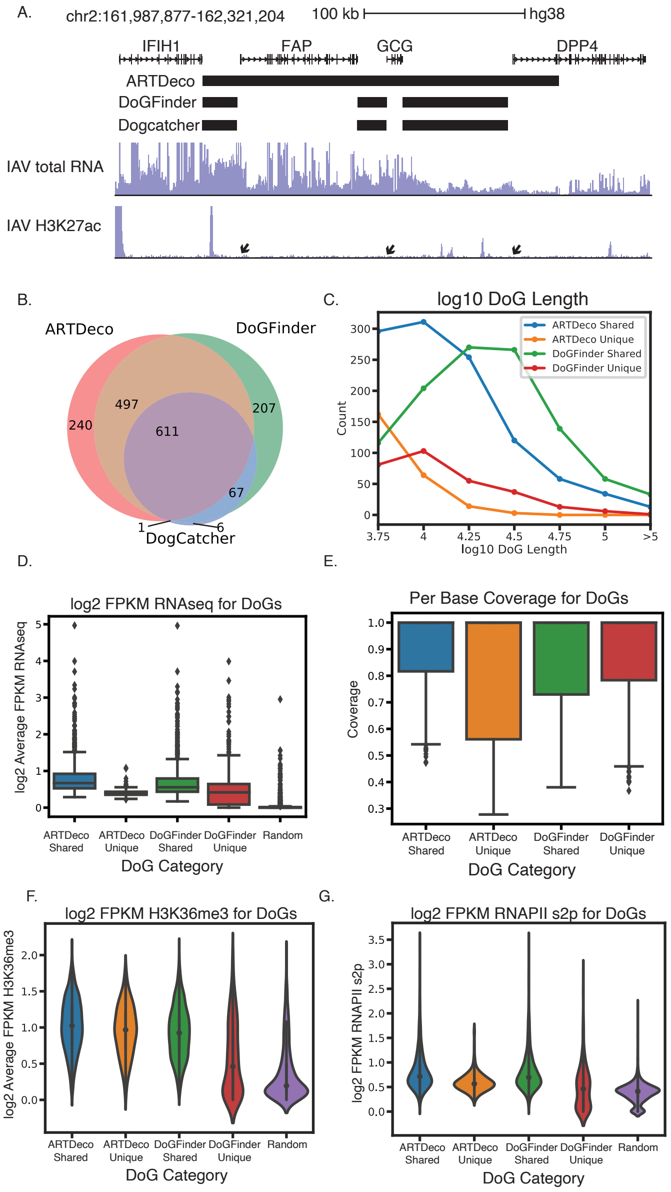
A.
chr2:161,987,877-162,321,204
100 kb
hg38
IFIH1
FAP
GCG
DPP4
ARTDeco
DoGFinder
Dogcatcher
IAV total RNA
IAV H3K27ac
B.
ARTDeco
DoGFinder
497
240
611
207
67
1
6
DogCatcher
C.
log10 DoG Length
ARTDeco Shared
ARTDeco Unique
DoGFinder Shared
DoGFinder Unique
Count
log10 DoG Length
D.
log2 FPKM RNAseq for DoGs
log2 Average FPKM RNAseq
ARTDeco Shared
ARTDeco Unique
DoGFinder Shared
DoGFinder Unique
Random
DoG Category
E.
Per Base Coverage for DoGs
Coverage
ARTDeco Shared
ARTDeco Unique
DoGFinder Shared
DoGFinder Unique
DoG Category
F.
log2 FPKM H3K36me3 for DoGs
log2 Average FPKM H3K36me3
ARTDeco Shared
ARTDeco Unique
DoGFinder Shared
DoGFinder Unique
Random
DoG Category
G.
log2 FPKM RNAPII s2p for DoGs
log2 FPKM RNAPII s2p
ARTDeco Shared
ARTDeco Unique
DoGFinder Shared
DoGFinder Unique
Random
DoG Category

# Figure S4

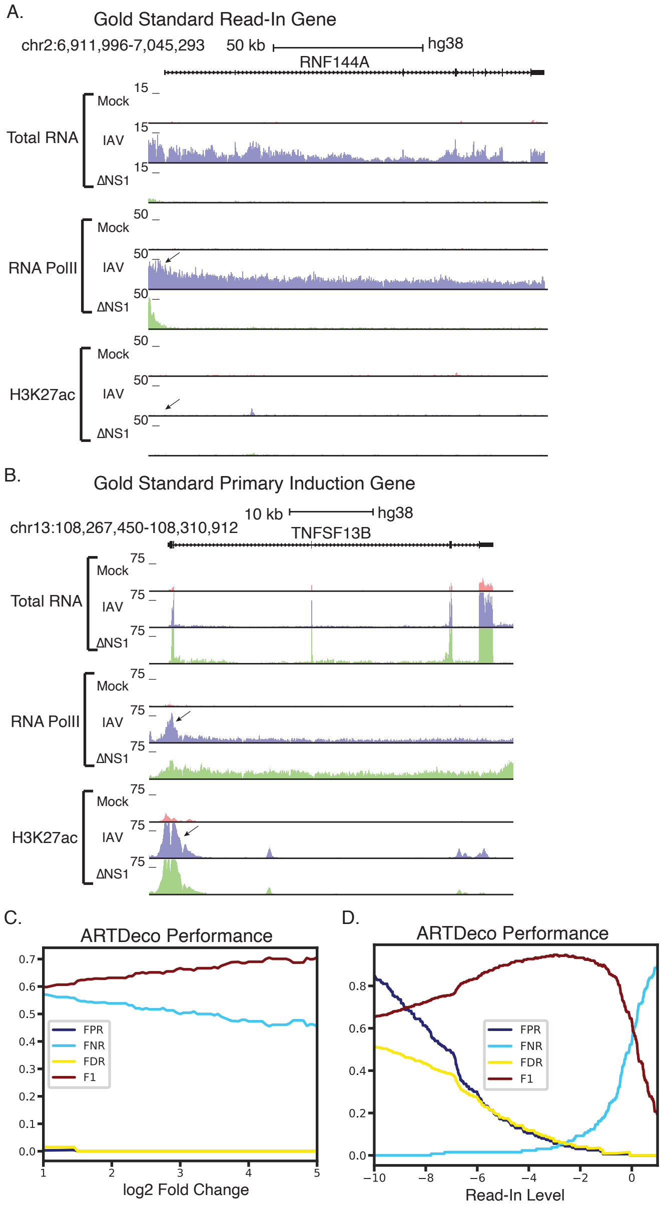
A.
Gold Standard Read-In Gene
chr2:6,911,996-7,045,293
50 kb
hg38
RNF144A
Total RNA
RNA PolII
H3K27ac
Mock
IAV
ΔNS1
15
50
B.
Gold Standard Primary Induction Gene
chr13:108,267,450-108,310,912
10 kb
hg38
TNFSF13B
75
C.
ARTDeco Performance
FPR
FNR
FDR
F1
log2 Fold Change
D.
ARTDeco Performance
Read-In Level

# Figure S5

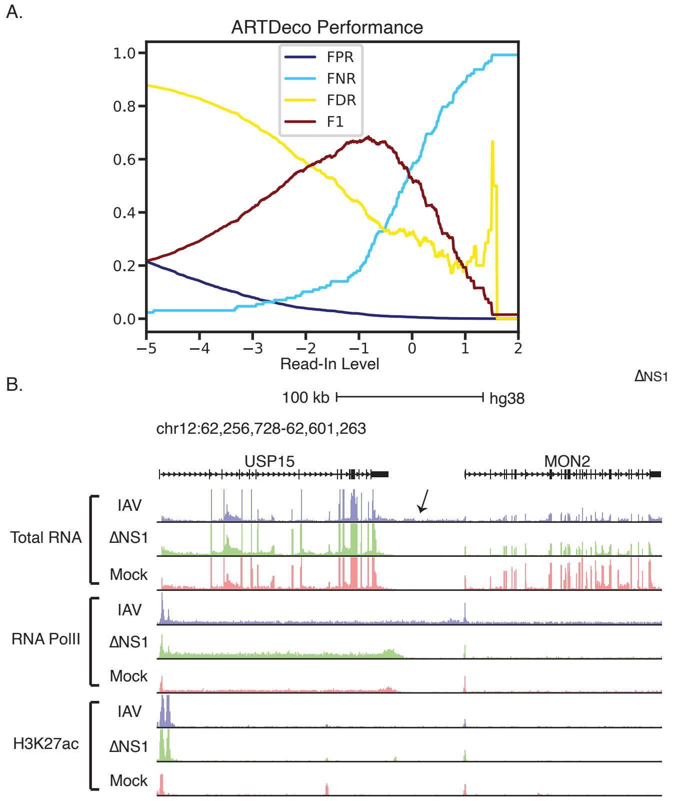
A.
ARTDeco Performance
FPR
FNR
FDR
F1
1.0
0.8
0.6
0.4
0.2
0.0
−5
−4
−3
−2
−1
0
1
2
Read-In Level
B.
ΔNS1
100 kb
hg38
chr12:62,256,728-62,601,263
USP15
MON2
Total RNA
RNA PolII
H3K27ac
IAV
ΔNS1
Mock

Figure S6

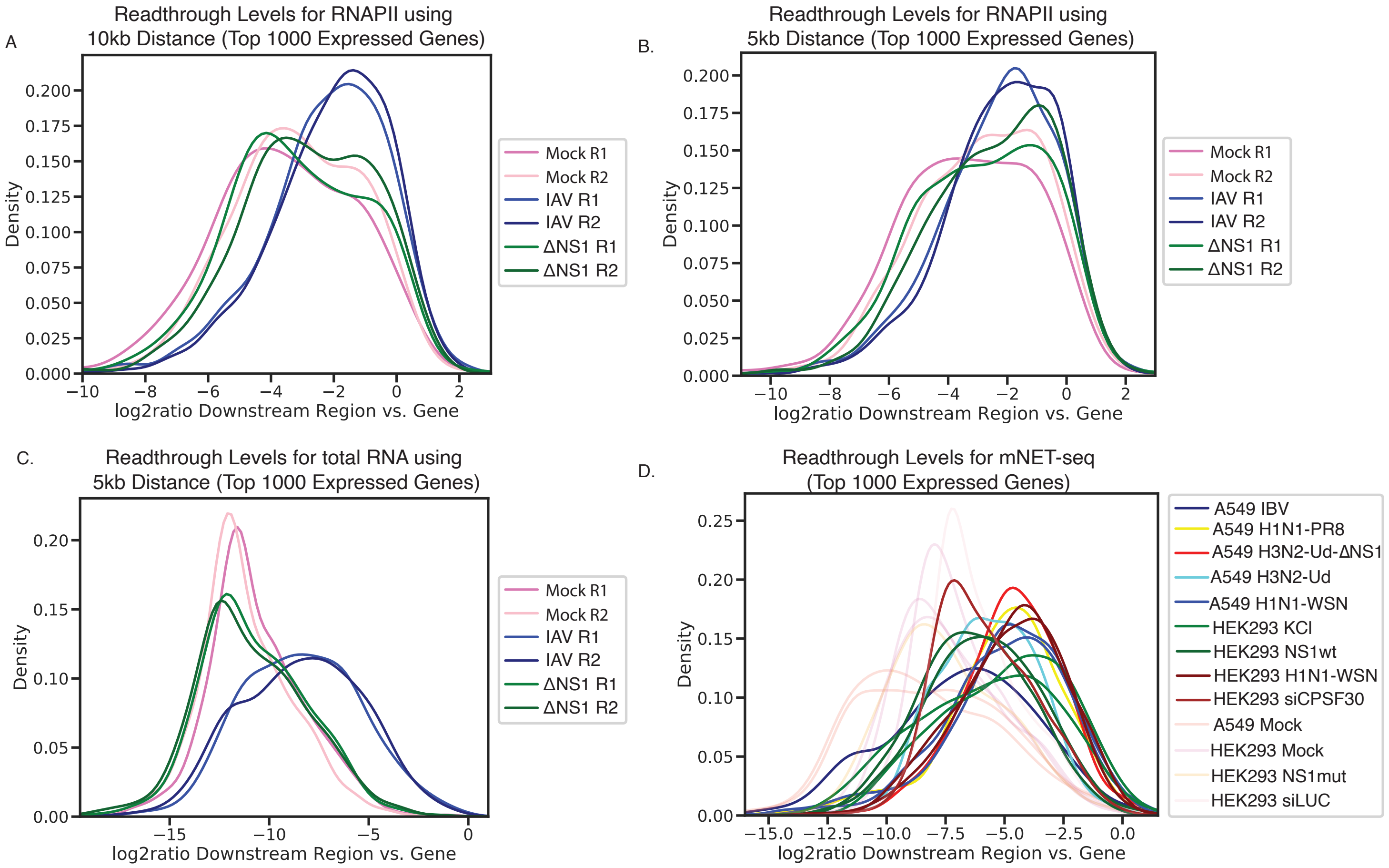
A
Readthrough Levels for RNAPII using
10kb Distance (Top 1000 Expressed Genes)
Density
log2ratio Downstream Region vs. Gene
Mock R1
Mock R2
IAV R1
IAV R2
ΔNS1 R1
ΔNS1 R2
B.
Readthrough Levels for RNAPII using
5kb Distance (Top 1000 Expressed Genes)
Density
log2ratio Downstream Region vs. Gene
Mock R1
Mock R2
IAV R1
IAV R2
ΔNS1 R1
ΔNS1 R2
C.
Readthrough Levels for total RNA using
5kb Distance (Top 1000 Expressed Genes)
Density
log2ratio Downstream Region vs. Gene
Mock R1
Mock R2
IAV R1
IAV R2
ΔNS1 R1
ΔNS1 R2
D.
Readthrough Levels for mNET-seq
(Top 1000 Expressed Genes)
Density
log2ratio Downstream Region vs. Gene
A549 IBV
A549 H1N1-PR8
A549 H3N2-Ud-ΔNS1
A549 H3N2-Ud
A549 H1N1-WSN
HEK293 KCl
HEK293 NS1wt
HEK293 H1N1-WSN
HEK293 siCPSF30
A549 Mock
HEK293 Mock
HEK293 NS1mut
HEK293 siLUC
