# Supplementary material for: ARTDeco: automatic readthrough transcription detection
Source: BMC Bioinformatics. 2020 May 26;21:214. doi: 10.1186/s12859-020-03551-0 (PMC7249449; doi:10.1186/s12859-020-03551-0)
Supplement: Supplementary file 5 — Additional file 5 Supplementary Methods. [file 12859_2020_3551_MOESM5_ESM.docx]

**Supplementary Methods**

**NGS data processing**

Data from Heinz et al. (2018) and Vilborg et al. (2017) were obtained from GEO accessions GSE103477 and GSE98906, respectively. Data from Bauer et al. (2018) was obtained from NCBI SRA SRP132032. Data from Quach et al. (2016) was obtained from the EGA accession EGAS00001001895. Reads from these data (and all data types therein) were trimmed using Cutadapt v2.4 (Martin 2011).  All RNA-seq data was aligned to reference genome using  STAR v. 2.7.0d (Dobin et al. 2013). RNA-seq data was either aligned to a combined genome of hg38 and Influenza A/Vietnam/1203/2004 (H5N1) HAlo, the mm10 genome, or a combined genome of hg38 and Influenza A/A/USSR/90/1977 (H1N1) for data from Heinz et al. (2018), Vilborg et al. (2017), and Quach et al. (2016), respectively. After alignment, RNA-seq data was processed by ARTDeco (detailed below). mNETseq data from Bauer et al. (2018) was aligned to the hg38 genome and aligned files were further processed using mNET_snr (Nojima et al. 2016) prior to ARTDeco processing. ChIP-seq and Start-seq data was from Heinz et al. (2018) was aligned to the hg38 genome using Bowtie2 v. 2.3.5 (Langmead and Salzberg 2012). Tag directories and peaks were called using HOMER v. 4.10 with the exception of RNAPII data which was processed by ARTDeco (detailed below) (Heinz et al. 2010). All NGS data were visualized on the UCSC genome browser using HOMER makeMultiWigHub.pl (Heinz et al. 2010; Kent 2002).

**ARTDeco data processing**

All RNA-seq data, RNAPII ChIPseq data from Heinz et al. (2018), and mNETseq data from Bauer et al. (2018) were run through the standard ARTDeco preprocessing and quantification (outlined above). GTF files from GENCODE (Frankish et al. 2019) were used as input (hg38 v28 for data mapped to hg38 [or a combined genome containing hg38 and viral genomes] and mm10 vM17 for data mapped to mm10). Only gene types in the categories protein_coding, lincRNA, bidirectional_promoter_lncRNA, and processed_transcript as defined by GENCODE were considered for read-in gene analysis. Chromosome sizes files were generated using Samtools (Li et al. 2009). For total RNA-seq data from Heinz et al. (2018) and Vilborg et al. (2017), read-in genes were called with expression of >0.25 FPKM and read-in ratios of > -1 when not using differential expression information. When using differential expression information, genes were considered upregulated if they had log2 fold change > , p-value < 0.05, and FPKM > 0.25. These were assigned as read-in genes if read-in levels were > -2 for Heinz et al. (2018) and > -1 for Vilborg et al. (2017) and if they fell into the above-mentioned gene categories. Thresholds were determined based upon benchmarking Heinz et al. (2018) data (described below). DoGs were called using default parameters for all datasets.

**Deconvolution of Gene Expression using Read-In Expression**

We took all upregulated genes as called by DESeq2 in the IAV condition relative to the mock condition and compared raw and corrected gene expression values in the IAV and ΔNS1 conditions. Similar to above, only gene types in the categories protein_coding, lincRNA, bidirectional_promoter_lncRNA, and processed_transcript as defined by GENCODE were considered.

**Benchmarking Read-In Gene Inference**

We curated a set of gold standard read-in and promoter-activated/primary induction genes using differential expression output from total RNA-seq from Heinz et al. (2018). As a positive control for identifying primary induction genes, we used data from samples infected with an IAV virus that expresses a truncated NS1 protein (ΔNS1) that does not cause readthrough transcription. We expected that genes considered upregulated in both IAV and ΔNS1 samples represent primary induction genes while genes upregulated in IAV samples but not ΔNS1 samples represent read-in genes. Differential expression analysis was carried out using DESeq2 (Love, Huber, and Anders 2014) as performed in the ARTDeco pipeline. Gold standard read-in genes were defined as true positives while gold standard promoter-activated/primary induction genes were defined as true negatives for performance evaluation. A gold-standard read-in gene was defined as being upregulated in IAV relative to ΔNS1 (log2 fold change > 2 and p < 0.05), expression in IAV > 0.25 FPKM, and expression in ΔNS1 < 0.5 FPKM while having no promoter-proximal H3K27ac or RNAPII ChIP-seq peaks. Promoter-activated genes (true negative when not using differential expression to infer read-in genes) were defined as having expression in IAV > 0.25 FPKM and having both H3K27ac and RNAPII ChIP-seq peaks near/on the promoter. Primary induction genes (true negative when using differential expression to infer read-in genes) were defined as upregulated in both IAV and ΔNS1 relative to the mock condition (log2 fold change > 2 and p < 0.05) with expression in both IAV and ΔNS1 above 0.25 FPKM and having promoter-proximal H3K27ac and RNAPII ChIP-seq peaks.

We computed various measures of performance such as false positive rate (FPR), false negative rate (FNR), false discovery rate (FDR) and F1 score. These were calculated as follows:

$$FPR=\frac{FP}{FP+TN}$$

$$FNR=\frac{FN}{FN+TP}$$

$$FDR=\frac{FP}{FP+TP}$$

$$F1=\frac{2*TP}{2*TP+FP+FN}$$

where true positives (TP) are correctly assigned read-in genes, false positives (FP) are incorrectly assigned read-in genes, true negatives (TN) are correctly assigned primary induction genes, and false negatives (FN) are incorrectly assigned primary induction genes. We then varied parameters such as log2 fold change, p-value, and read-in level to test the ability of ARTDeco to infer read-in genes both with and without differential expression information included.

**Functional analysis of read-in genes**

Read-in genes were inferred using differential expression as described above for both the Heinz et al. (2018) and Vilborg et al. (2017) data. Gene ontology (GO) enrichment was performed using GOATOOLS (Klopfenstein et al., 2018) on read-in and primary induction genes. Additionally, motif enrichment of promoters was performed using HOMER (Heinz et al., 2010).

**DoGFinder and Dogcatcher DoG Comparison**

DoGFinder (Wiesel, Sabath, and Shalgi 2018) and Dogcatcher (Melnick et al., 2019) were run in “window mode” with a window of 500 bp and coverage of 0.6 using both IAV replicates. DoGs discovered using DoGFinder in each replicate were combined using the Union_DoGs_annotation function. DoGs discovered using Dogcatcher were combined using the 2.5_Dogcatcher_filter.py

script. The characteristics of DoGs discovered by DoGFinder (i.e., identity, length, epigenomic signatures of transcription elongation) were compared to the set of combined ARTDeco DoGs for both IAV replicates in order to assess similarities and differences in transcript detection. Random DoG regions were generated using bedtools to shuffle genomic locations of DoGs discovered by ARTDeco in IAV replicates (Quinlan and Hall 2010). Per base coverage of DoGs was computed using bedtools coverage (Quinlan and Hall, 2010).

**DoGFinder and Dogcatcher Runtime Comparison**

DoGFinder, Dogcatcher and ARTDeco were each run 10 times on mock, IAV, and ΔNS1 in order to assess runtime. All runs were performed on 50 Intel Xeon E5-2697 v3 @ 2.60GHz CPUs. DoGFinder was run in two stages. First, preprocessing was performed on these BAM files in order to ensure proper formatting for DoGFinder and Dogcatcher. A Snakemake workflow (Koster and Rahmann 2012) that combined custom scripts and Samtools (Li et al. 2009) was implemented for both DoGFinder and Dogcatcher to convert the BAM files to SAM files, switch strand orientation, sort the SAM files, and index the resulting BAM files. BAM files were converted into bedGraphs using bedtools (Quinlan et al., 2010) for Dogcatcher preprocessing in addition to the above steps. Then, DoGFinder was performed as detailed above with the addition of generating expression data for each set of DoGs discovered for each experiment as well as all DoGs (as discovered by Union_DoGs_annotation) using the Get_DoGs_rpkm function. ARTDeco was run as described above in both full mode (i.e., using all functions including read-in gene inference and differential expression) and in DoG discovery mode. Dogcatcher was run with and without differential expression (i.e., including or excluding the following scripts 3.0_Create_R_subread_DESeq2_script.py, 4.0_Dogcatcher_Rsubread_DESeq2.py, and 5.0_filter_sig_DESeq2.py.

**Supplementary References**

Bauer, David L. V., Michael Tellier, Mónica Martínez-Alonso, Takayuki Nojima, Nick J. Proudfoot, Shona Murphy, and Ervin Fodor. 2018. “Influenza Virus Mounts a Two-Pronged Attack on Host RNA Polymerase II Transcription.” *Cell Reports* 23 (7): 2119–29.e3.

Dobin, Alexander, Carrie A. Davis, Felix Schlesinger, Jorg Drenkow, Chris Zaleski, Sonali Jha, Philippe Batut, Mark Chaisson, and Thomas R. Gingeras. 2013. “STAR: Ultrafast Universal RNA-Seq Aligner.” *Bioinformatics*  29 (1): 15–21.

Frankish, Adam, Mark Diekhans, Anne-Maud Ferreira, Rory Johnson, Irwin Jungreis, Jane Loveland, Jonathan M. Mudge, et al. 2019. “GENCODE Reference Annotation for the Human and Mouse Genomes.” *Nucleic Acids Research* 47 (D1): D766–73.

Heinz, Sven, Christopher Benner, Nathanael Spann, Eric Bertolino, Yin C. Lin, Peter Laslo, Jason X. Cheng, Cornelis Murre, Harinder Singh, and Christopher K. Glass. 2010. “Simple Combinations of Lineage-Determining Transcription Factors Prime Cis-Regulatory Elements Required for Macrophage and B Cell Identities.” *Molecular Cell* 38 (4): 576–89.

Heinz, Sven, Lorane Texari, Michael G. B. Hayes, Matthew Urbanowski, Max W. Chang, Ninvita Givarkes, Alexander Rialdi, et al. 2018. “Transcription Elongation Can Affect Genome 3D Structure.” *Cell* 174 (6): 1522–36.e22.

Kent, W. J. 2002. “The Human Genome Browser at UCSC.” *Genome Research*. <https://doi.org/10.1101/gr.229102>.

Klopfenstein, D. V., Liangsheng Zhang, Brent S. Pedersen, Fidel Ramírez, Alex Warwick Vesztrocy, Aurélien Naldi, Christopher J. Mungall, et al. 2018. “GOATOOLS: A Python Library for Gene Ontology Analyses.” Scientific Reports 8 (1): 10872.

Koster, J., and S. Rahmann. 2012. “Snakemake--a Scalable Bioinformatics Workflow Engine.” *Bioinformatics*. https://doi.org/10.1093/bioinformatics/bts480[.](http://paperpile.com/b/qclzem/P9Dw)

Langmead, Ben, and Steven L. Salzberg. 2012. “Fast Gapped-Read Alignment with Bowtie 2.” *Nature Methods* 9 (4): 357–59.

Li, Heng, Bob Handsaker, Alec Wysoker, Tim Fennell, Jue Ruan, Nils Homer, Gabor Marth, Goncalo Abecasis, Richard Durbin, and 1000 Genome Project Data Processing Subgroup. 2009. “The Sequence Alignment/Map Format and SAMtools.” *Bioinformatics*  25 (16): 2078–79.

Love, Michael I., Wolfgang Huber, and Simon Anders. 2014. “Moderated Estimation of Fold Change and Dispersion for RNA-Seq Data with DESeq2.” *Genome Biology* 15 (12): 550.

Martin, Marcel. 2011. “Cutadapt Removes Adapter Sequences from High-Throughput Sequencing Reads.” *EMBnet.journal*. https://doi.org/10.14806/ej.17.1.200[.](http://paperpile.com/b/qclzem/wkd2S)

Nojima, Takayuki, Tomás Gomes, Maria Carmo-Fonseca, and Nicholas J. Proudfoot. 2016. “Mammalian NET-Seq Analysis Defines Nascent RNA Profiles and Associated RNA Processing Genome-Wide.” *Nature Protocols* 11 (3): 413–28.

Quach, Hélène, Maxime Rotival, Julien Pothlichet, Yong-Hwee Eddie Loh, Michael Dannemann, Nora Zidane, Guillaume Laval, et al. 2016. “Genetic Adaptation and Neandertal Admixture Shaped the Immune System of Human Populations.” *Cell* 167 (3): 643–56.e17.

Quinlan, Aaron R., and Ira M. Hall. 2010. “BEDTools: A Flexible Suite of Utilities for Comparing Genomic Features.” *Bioinformatics*. <https://doi.org/10.1093/bioinformatics/btq033>[.](http://paperpile.com/b/qclzem/CRz1u)

Vilborg, Anna, Niv Sabath, Yuval Wiesel, Jenny Nathans, Flonia Levy-Adam, Therese A. Yario, Joan A. Steitz, and Reut Shalgi. 2017. “Comparative Analysis Reveals Genomic Features of Stress-Induced Transcriptional Readthrough.” *Proceedings of the National Academy of Sciences* 114 (40): E8362–71.

Wang, Liguo, Shengqin Wang, and Wei Li. 2012. “RSeQC: Quality Control of RNA-Seq Experiments.” *Bioinformatics*  28 (16): 2184–85.

Wiesel, Yuval, Niv Sabath, and Reut Shalgi. 2018. “DoGFinder: A Software for the Discovery and Quantification of Readthrough Transcripts from RNA-Seq.” *BMC Genomics* 19 (1): 597.
